# Rapid directed molecular evolution of fluorescent proteins in mammalian cells

**DOI:** 10.1002/pro.4261

**Published:** 2021-12-30

**Authors:** Siranush Babakhanova, Erica E. Jung, Kazuhiko Namikawa, Hanbin Zhang, Yangdong Wang, Oksana M. Subach, Dmitry A. Korzhenevskiy, Tatiana V. Rakitina, Xian Xiao, Wenjing Wang, Jing Shi, Mikhail Drobizhev, Demian Park, Lea Eisenhard, Hongyun Tang, Reinhard W. Köster, Fedor V. Subach, Edward S. Boyden, Kiryl D. Piatkevich

**Affiliations:** ^1^ Media Arts and Sciences Massachusetts Institute of Technology (MIT) Cambridge Massachusetts USA; ^2^ Department of Physics MIT Cambridge Massachusetts USA; ^3^ Department of Electrical Engineering and Computer Science MIT Cambridge Massachusetts USA; ^4^ MIT McGovern Institute for Brain Research, MIT Cambridge Massachusetts USA; ^5^ Department of Mechanical and Industrial Engineering The University of Illinois at Chicago Chicago Illinois USA; ^6^ Division of Cellular and Molecular Neurobiology, Zoological Institute Technische Universität Braunschweig Braunschweig Germany; ^7^ School of Life Sciences Westlake University Zhejiang Hangzhou China; ^8^ Westlake Laboratory of Life Sciences and Biomedicine Zhejiang Hangzhou China; ^9^ Institute of Basic Medical Sciences Westlake Institute for Advanced Study Zhejiang Hangzhou China; ^10^ National Research Center “Kurchatov Institute” Moscow Russian Federation; ^11^ Shemyakin‐Ovchinnikov Institute of Bioorganic Chemistry, RAS Moscow Russian Federation; ^12^ Department of Microbiology and Immunology Montana State University Bozeman Montana USA; ^13^ Department of Biological Engineering MIT Cambridge Massachusetts USA; ^14^ Department of Brain and Cognitive Sciences MIT Cambridge Massachusetts USA; ^15^ Howard Hughes Medical Institute Cambridge Massachusetts USA; ^16^ Koch Institute MIT Cambridge Massachusetts USA

**Keywords:** directed molecular evolution, fluorescent proteins, in vivo fluorescence imaging, neuroimaging, optogenetics

## Abstract

In vivo imaging of model organisms is heavily reliant on fluorescent proteins with high intracellular brightness. Here we describe a practical method for rapid optimization of fluorescent proteins via directed molecular evolution in cultured mammalian cells. Using this method, we were able to perform screening of large gene libraries containing up to 2 × 10^7^ independent random genes of fluorescent proteins expressed in HEK cells, completing one iteration of directed evolution in a course of 8 days. We employed this approach to develop a set of green and near‐infrared fluorescent proteins with enhanced intracellular brightness. The developed near‐infrared fluorescent proteins demonstrated high performance for fluorescent labeling of neurons in culture and in vivo in model organisms such as *Caenorhabditis elegans*, *Drosophila*, zebrafish, and mice. Spectral properties of the optimized near‐infrared fluorescent proteins enabled crosstalk‐free multicolor imaging in combination with common green and red fluorescent proteins, as well as dual‐color near‐infrared fluorescence imaging. The described method has a great potential to be adopted by protein engineers due to its simplicity and practicality. We also believe that the new enhanced fluorescent proteins will find wide application for in vivo multicolor imaging of small model organisms.

## 1 INTRODUCTION

Fluorescent proteins (FPs) became indispensable tools for in vivo imaging of cellular and subcellular structures in model organisms.^1^ Since the cloning of the first green FP from jellyfish *Aequorea victoria* in 1992,^2^ a myriad of chromoproteins with various spectral and biochemical properties have been cloned from diverse natural sources such as corals,^3^ fish,^4^ plants,^5^ soil bacteria,^6^ and cyanobacteria.^7^ However, all naturally occurring chromoproteins have to be modified, optimized, or even reengineered in order to be utilized for fluorescence imaging in vivo.^8^, ^9^, ^10^, ^11^, ^12^ Among all biochemical characteristics, intracellular brightness is one of the most crucial properties for *in vivo* applications that protein engineers and developers choose to optimize before everything else.^13^, ^14^ FPs are usually optimized via directed molecular evolution by iteratively generating and screening large gene libraries in bacterial cells.^13^, ^14^, ^15^ However, high molecular brightness, commonly screened for in bacterial cells, does not always correspond to high intracellular brightness when expressed in cultured mammalian cells^8^, ^9^, ^16^ or in vivo.^16^, ^17^, ^18^ This phenomenon is particularly well documented for bacteriophytochrome‐based FPs,^16^, ^19^, ^20^ whose fluorescence relies on the incorporation of the chromophore biliverdin (BV) from the bulk.^21^ Ideally, the development of functional proteins for *in vivo* imaging should be performed in an environment physiologically relevant to the final hosts to ensure proper protein folding, localization, and posttranslational modification.^22^, ^23^ Yeasts, although eukaryotic cells, which provide convenience of large gene library expression like bacteria, may not serve as an ideal host system for FPs as it was shown that brightness in yeast cells is not necessarily retained in mammalian cells.^24^ In this regard, vertebrate cell lines represent a promising expression host for directed molecular evolution of FPs. Indeed, several studies demonstrated the possibility to evolve FPs in cultured chicken^25^ or mammalian cells.^26^, ^27^, ^28^ However, the proposed methods did not find wide adaptation among protein engineers due to several limitations and drawbacks. First, all previously developed methods for directed molecular evolution in cultured cells from vertebrates involve establishing cell lines stably maintaining target genes, in a way where any given cell expresses ideally no more than one copy of a target gene.^26^, ^27^, ^28^ However, both commonly used single gene copy delivery methods, such as electroporation and retroviral transduction, and establishing stable cell lines, are time‐consuming and laborious, and complicated by apoptosis, low efficiency of stable gene integration, and long cell doubling time. Second, in situ diversification of target genes using a cytidine deaminase^25^, ^26^, ^29^ or viral replication^30^ has a low mutation rate of only 1–3 mutations per kilobase pair in comparison to 9–16 mutations per kilobase pair for regular error‐prone PCR typically used for FP development. Higher mutation rates can be achieved with CRISPR/Cas9 editing technology^28^ or via in vitro mutagenesis,^27^ but comes with a limited library size of 10^5^–10^6^ independent clones. Third, recovery of target genes after screening and sorting is typically done from large pools (10^3^–10^7^) of collected cells with subsequent random picking of just a few (10–200) clones from the pool that significantly reduces the chance of finding the best variant.^26^, ^27^, ^28^ As a result, the previously reported methods for evolution of FPs in mammalian cells were not adopted by protein engineers and the developed FPs did not find wide utilization for in vivo imaging.

Here, we describe a simple and practical method that overcomes major limitations and drawbacks of currently available methods for directed molecular evolution of FPs in mammalian cells. The developed method allowed us to perform optimization of intracellular brightness in the course of 4 days per iteration by generation and screening of large (up to 2 × 10^7^) and highly diverse (10–15 mutations per kilobase pair) libraries of FPs in mammalian cells, and an extra 3 days for isolation and identification of individual variants. Moreover, our method allows rapid target gene recovery from very small pools of collected cells (10–100 cells), enabling genotyping and detailed phenotyping of rare cell populations common for large random libraries. We employed the described method to develop a set of green and near‐infrared (NIR) FPs, named phiLOV3, TagRFP658, and miRFP2, with enhanced intracellular brightness. The improvements of intracellular brightness for phiLOV3 and miRFP2 did not correlate with molecular brightness when compared to respective parental proteins, thus highlighting the importance of protein optimization in a specific cellular context. Using rational design, we generated an enhanced version of miRFP2 with greatly increased intracellular brightness, called emiRFP2. We demonstrated applicability of the developed proteins for in vivo imaging of neurons in *Caenorhabditis elegans*, *Drosophila*, zebrafish, and intact mouse brain tissue. Combination of the developed FPs with other common green and red FPs enabled crosstalk‐free multicolor imaging using standard optical configurations. Moreover, we performed dual‐color NIR imaging of subcellular structures using a combination of emiRFP2 with blue‐shifted NIR FP mCardinal. The developed method has a great potential to be adopted by protein engineers for optimization of FPs in mammalian cells. We also believe that the new enhanced FPs will find wide application for in vivo imaging of small model organisms.

## 2 RESULTS

### 2.1 Protein optimization in mammalian cells

**FIGURE 1:**
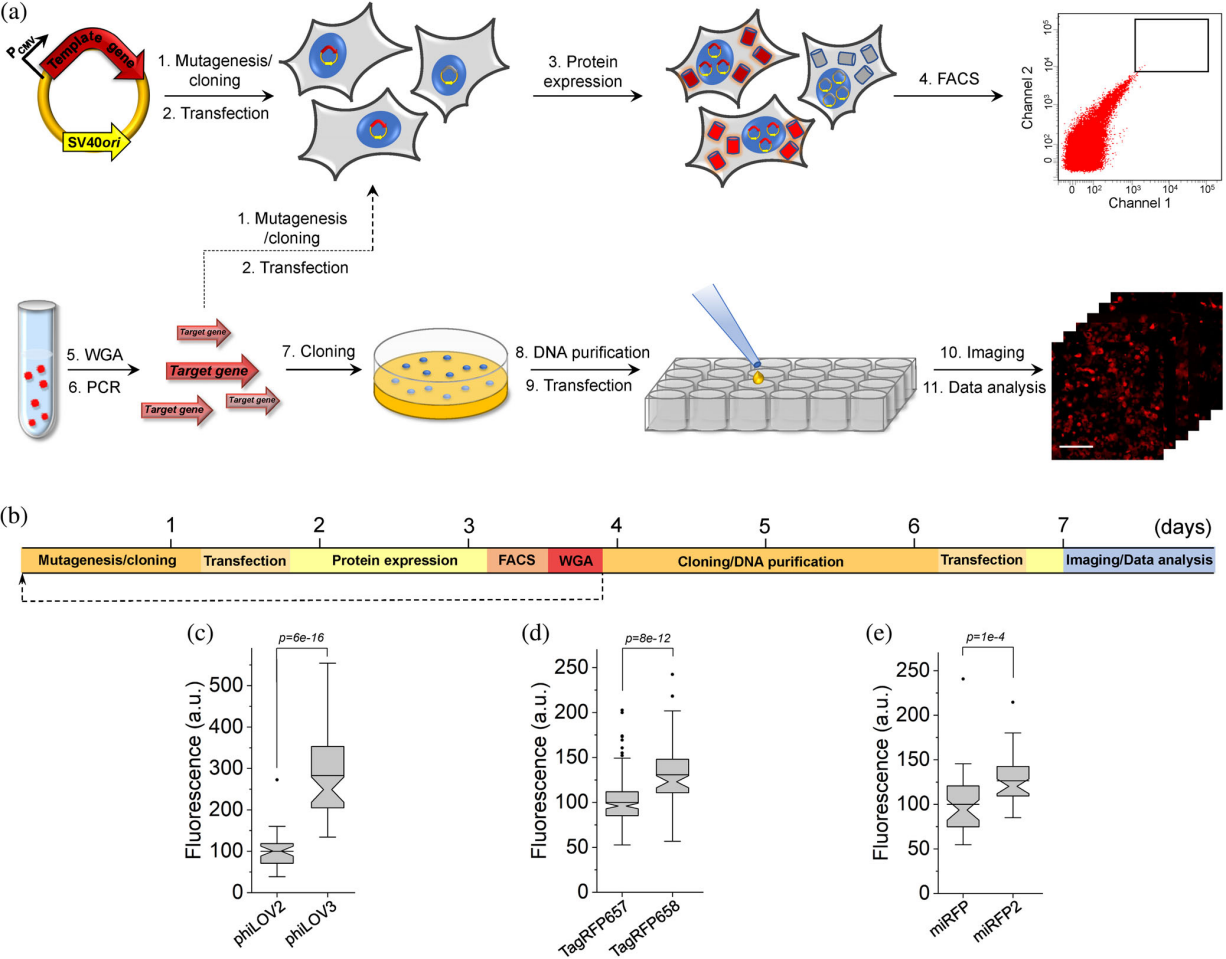
Rapid directed molecular evolution of fluorescent proteins in mammalian cells. (a) Workflow for directed evolution of proteins in HEK293FT cells using single‐gene delivery via modified calcium phosphate transfection, fluorescence‐activating cell sorting (FACS), and automated fluorescence imaging; WGA, whole‐genome amplification. (b) Timeline of directed molecular evolution. (c) Green fluorescence of phiLOV2 and phiLOV3 expressed in HEK293FT cells (*n* = 40 and 59 cells, respectively, from two independent transfections for each protein; one‐way analysis of variance [ANOVA] is used throughout this figure). Imaging conditions: excitation 475/34 nm from an LED, emission 527/50 nm. Box plots with notches are used throughout this paper, when *n* > 9 (the narrow part of notch, median; top and bottom of the notch, 95% confidence interval for the median; top and bottom horizontal lines, 25 and 75% percentiles for the data; whiskers extend 1.5× the interquartile range from the 25th and 75th percentiles; horizontal line, mean; dots represent outliers, data points which are less than the 25th percentile or greater than the 75th percentile by more than 1.5 times the interquartile range). (d) Near‐infrared fluorescence of TagRFP657 and TagRFP658 expressed in HEK293FT cells (*n* = 181 and 76 cells, respectively, from two independent transfections for each protein). Imaging conditions: excitation 631/28 nm from an LED, emission 664LP. (e) Near‐infrared fluorescence of miRFP and miRFP2 expressed in HEK293FT cells (*n* = 40 and 41 cells, respectively, from two independent transfections for each protein). Imaging conditions as in (d)

The rapid protein optimization via directed molecular evolution is based on a simple and scalable method for expression of large gene libraries in mammalian cells in combination with high‐throughput live cell screening techniques, for example, fluorescence‐activated cell sorting (FACS). The workflow includes six major hands‐on steps: (a) preparation of gene library; (b) transfection of mammalian cells with gene library in bulk; (c) screening and collection of individual cells; (d) cloning of the target genes from selected cells into expression vector; (e) transfection of the cloned plasmids into mammalian cells; and (f) imaging and selection of individual clones (Figure 1a). One iteration of directed molecular evolution can be carried out in about 8 days (Figure 1b). In the case of the bulk selection, when pools of selected genes are subjected to the next round of evolution, mutagenesis and screening can be performed with a period of ~3.5 days (Figure 1b). To validate the proposed approach, we decided to enhance intracellular brightness of the biochemically and spectrally distinct FPs. We chose a set of FPs that originated from the four evolutionary different classes of chromoproteins and those that are diverse in their later synthetic evolution conditions. Namely, we chose a green FP phiLOV2.1 engineered from the flavin‐binding light, oxygen, or voltage (LOV) sensing domain,^31^ a naturally occurring green FP UnaG cloned from freshwater eel *Anguilla japonica*,^4^ an engineered far‐red fluorescent GFP‐like FP TagRFP657,^32^ and an NIR FP miRFP derived from PAS‐GAF domains of the *Rp*BphP1 bacteriophytochrome.^33^ By selecting already optimized FPs as templates, our goal was to demonstrate the great potential of the proposed approach to further enhance desired characteristics in mammalian cells. First, human‐codon optimized genes of the selected proteins were subjected to error‐prone PCR and cloned into the mammalian expression vector containing an SV40 origin of replication. The SV40 origin of replication significantly increases the expression levels of transgene under transient transfection conditions due to episomal replication of the plasmid within a host cell that expresses the SV40 large T‐antigen,^34^ which is crucial under one plasmid per cell transfection conditions. The generated random libraries, containing around 10^6^–10^7^ independent clones, were transfected into HEK293FT cells using the modified calcium phosphate method, which was optimized for single gene copy per cell delivery.^35^ After plasmid replication and protein expression for 48 hr, we used FACS to collect ~100 cells with the highest fluorescence intensity for each library. Next, the target genes, recovered from the pools of collected cells, were either subjected to the next round of directed evolution or directly cloned into the mammalian expression vector (Figure 1a). In the latter case, several hundreds of randomly picked clones were individually transfected into HEK cells to compare their brightness to the corresponding parental proteins. Overall, one iteration of directed evolution was carried out within ~3.5 days and additional ~4 days were required to clone, express, assess, and sequence individual mutants (Figure 1b). We carried out two rounds of directed evolution for each template followed by screening of individually picked clones expressed in HEK cells under fluorescence microscope (see Supplementary Table 1 for the screening conditions and parameters). Assessment of the clones selected from the UnaG library did not identify variants with improved intracellular brightness, although sequencing of the brightest selected variants revealed amino acid substitutions in the structurally important regions (Supplementary Figures 1 and 2). Therefore, we did not perform further characterization of the UnaG mutants. For phiLOV2.1 and TagRFP657, we identified multiple variants that outperformed corresponding parental proteins in terms of intracellular brightness and in case of miRFP, we identified only one mutant with improved brightness (Supplementary Figure 1). We selected the brightest variant from each library for further evaluation. However, during imaging we found out that the brightest TagRFP657 variant had very limited photostability, we decided to choose the second brightest variant, which was confirmed to have higher than the parental protein photostability (Supplementary Figure 1). To confirm that the observed improvements were statistically significant, we repeated the measurements for each selected variant on several biological replicates in HEK cells. Indeed, all variants showed biologically and statistically significant improvements in intracellular brightness. The phiLOV2.1 variant showed 2.8‐fold higher brightness over parental protein, the TagRFP657 and miRFP variants showed 30 and 27% over parental proteins, respectively (Figure 1c–e). Sequence analysis of the selected variants revealed two amino acid mutations in phiLOV2.1, six in TagRFP657, and nine in miRFP (Supplementary Figures 3–5). Correspondingly, we named the identified variants as phiLOV3, TagRFP658, and miRFP2, and used them for further detailed characterization. As a result, we were able to complete two iterations of directed molecular evolution in mammalian cells within 8 days enhancing intracellular brightness of the selected FPs (Figure 1).

### 2.2 Spectral and biochemical characterization of the optimized FPs

**FIGURE 2:**
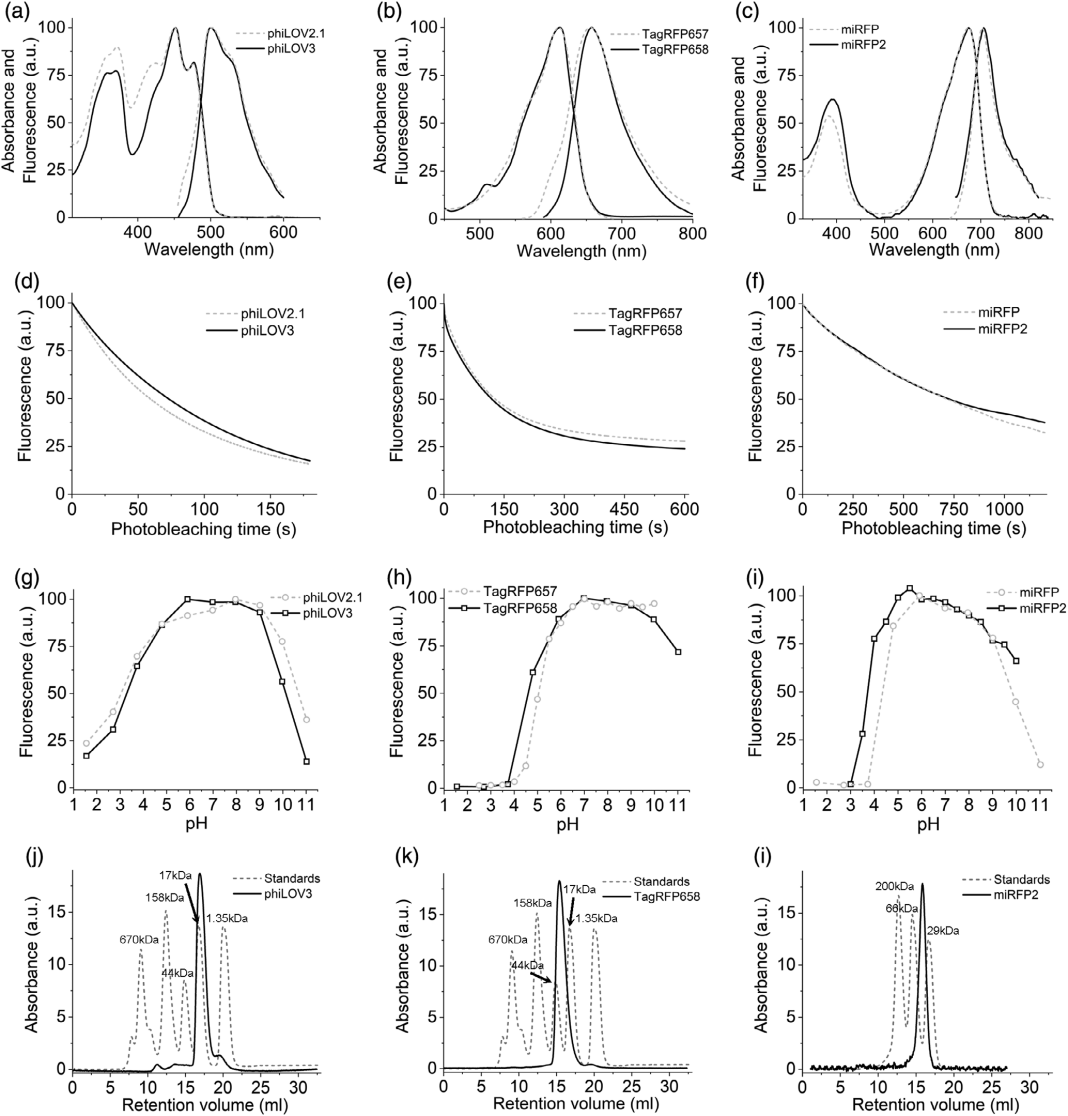
Spectroscopic and biochemical characterization of phiLOV3, TagRFP658, and miRFP2 in comparison to their precursors. (a–c) Absorption and fluorescence emission spectra of (a) phiLOV2.1 (dashed line) and phiLOV3 (solid line), (b) TagRFP657 (dashed line) and TagRFP658 (solid line), and (c) miRFP (dashed line) and miRFP2 (solid line). (d) Photobleaching curves of phiLOV2.1 (dashed line) and phiLOV3 (solid line) expressed in HEK293FT cells (*n* = 18 cells from 1 transfected sample, each). Photobleaching conditions: excitation 475/34 nm from an LED at ~5 mW/mm^2^. (e) Photobleaching curves of TagRFP657 (dashed line) and TagRFP658 (solid line) expressed in HEK293FT cells (*n* = 16 cells from 1 transfected sample, each). Photobleaching conditions: excitation 628/31BP from an LED at 95 mW/mm^2^. (f) Photobleaching curves of miRFP (dashed line) and miRFP2 (solid line) expressed in primary cultured mouse neurons (*n* = 25 and 61 cells, respectively, from one culture, each). Photobleaching conditions: excitation 628/31BP from an LED at 88 mW/mm^2^. (g–i) pH dependence of fluorescence for (g) phiLOV2.1 (dashed line) and phiLOV3 (solid line), (h) TagRFP657 (dashed line; data from Reference 32) and TagRFP658 (solid line), and (i) miRFP (dashed line; data from Reference 35) and miRFP2 (solid line). (j–l) Size‐exclusion chromatography of (j) phiLOV3 at a concentration of 3.8 mg/ml, (k) TagRFP658 at a concentration of 8 mg/ml, and (l) miRFP2 at a concentration of 8.8 mg/ml (solid lines) and the indicated molecular weight standards (dashed lines)

The introduced mutations did not alter the spectral properties of the developed proteins when compared to the corresponding parental proteins, showing only insignificant shifts in the maxima of the major bands (Figure 2a–c, Tables 1 and 2). Measured in solution using proteins purified from *Escherichia coli*, the molecular brightness of phiLOV3 and TagRFP658 was about 29 and 21% higher than that of their precursors, respectively, while molecular brightness of miRFP2 was 3.7‐fold lower than that of miRFP (Tables 1 and 2). Improvement of the TagRFP568 intracellular brightness corresponded to increase in molecular brightness over its precursor. However, relative intracellular brightness of phiLOV3 and miRFP2 in HEK cells was several fold higher than their molecular brightness when compared to phiLOV2.1 and miRFP, respectively (Tables 1 and 2). The TagRFP658 and miRFP2 proteins exhibited similar intracellular photostability compared to the corresponding parental proteins, while phiLOV3 showed about 23% improvement in photostability halftime over its precursor (Figure 2d–f). The pH‐stability of fluorescence for phiLOV3, TagRFP658, and miRFP2 was characterized by p*K*_a_ values of 3.3, 4.6, and 3.7, respectively, which were similar to that of the corresponding parental proteins (Figure 2g–i, Tables 1 and 2). Size‐exclusion chromatography demonstrated that the developed proteins preserved monomeric state even at a high concentration in solution (Figure 2j–l, see Supplementary Figure 6 for calibration plots). We also measured two‐photon cross section for TagRFP658 to explore its utility for two‐photon microscopy. TagRFP658 exhibited a major peak at 1230 nm in two‐photon spectrum coinciding with one‐photon peak (at double wavelength) rather well and exhibited a strong feature in the region corresponding to the S_0_ → S_n_ transitions with strong absorption only at wavelength below 830 nm (Supplementary Figure 7).

**TABLE 1:** Properties of the FMN‐binding phiLOV2.1 and phiLOV3 FPs

Protein	Abs (nm)	Em (nm)	EC (M^−1^ cm^−1^)	QY (%)	Molecular brightness^a^	p*K*_a_	Brightness in HEK cells (%)^b^	Intracellular photostability (s)^c^
phiLOV2.1	451, 476	501	13,500	20	2,700	3.0	100	59
phiLOV3	452, 477	502	15,800	22	3,480	3.3	283	73

Abbreviations: Abs, absorbance peak; Em, fluorescence emission peak; EC, extinction coefficient; ND, not determined; QY, quantum yield.

^a^ Molecular brightness is a product of extinction coefficient and fluorescence emission QY.

^b^ Determined as mean green fluorescence relative to phiLOV2.1.

^c^ Measured under continuous 470/25 nm wide‐filed illumination.

**TABLE 2:** Spectral and biochemical properties of the selected NIR FPs

Protein	Abs (nm)	Em (nm)	EC (M^−1^ cm^−1^)	QY (%)	Molecular brightness^a^	p*K*_a_	Brightness in HEK cells (%)^b^	Photostability (s)^c^	Initial photobleaching rate (%/s)^d^	Ref
mCardinal	603	651	79,000	18	14,220	5.3	290	340	0.24	^63^
mMaroon1	609	657	80,000	11	8,800	6.2	125^e^	ND	ND	^41^
iRFP670	643	670	114,000	11	15,540	4.0	154	245	0.56	^37^
miRFP670nano	645	670	95,000	10.8	10,260	3.7	8^e^	ND	ND	^19^
miRFP680	661	680	94,000	14.5	13,630	4.5	160	ND	0.06	^20^
iRFP682	663	682	90,000	11	9,900	4.5	140	904	0.09	^37^
miRFP703	674	703	90,900	8.6	7,820	4.5	48	ND	ND	^36^
mRhubard713	690	713	113,500	7.6	8,630	ND	33	ND	0.02	^38^
miRFP720	702	720	98,000	6.1	5,980	4.5	61	ND	0.03	^39^
TagRFP657	611	657	34,000	10	3,400	5.0	61^f^	ND	ND	^32^
TagRFP658	611	658	41,200	10	4,120	4.6	79	640	0.12	This study
miRFP	674	703	92,400	9.7	8,960	4.3	79^g^	ND	ND	^35^
miRFP2	676	706	55,600	4.3	2,390	3.7	100	995	0.08	This study
emiRFP2	ND	ND	ND	ND	ND	ND	205	1,050	0.08	This study

Abbreviations: Abs, absorbance peak; Em, fluorescence emission peak; EC, extinction coefficient; FPs, fluorescent proteins; ND, not determined; NIR, near‐infrared; QY, quantum yield.

^a^ Molecular brightness is a product of extinction coefficient and fluorescence emission QY.

^b^ Determined as a Cy5‐to‐green fluorescence intensity ratio for live HEK cells relative to miRFP2 unless otherwise state.

^c^ Measured in live HEK cells under continuous wide‐field illumination with 631 nm laser at 66 mW/mm^2^.

^d^ Determined as a liner fit of the initial segment of photobleaching curve.

^e^ Determined as mean Cy5 fluorescence intensity in live HEK cells relative to mCardinal.

^f^ Determined as mean Cy5 fluorescence intensity in live HEK cells relative to TagRFP658.

^g^ Determined as mean Cy5 fluorescence intensity in live HEK cells relative to miRFP2.

To investigate the significant difference in relative brightness of miRFP and miRFP2 in vitro and in cell culture, we evaluated BV binding efficiency in HEK cells by measuring intracellular brightness under different concentrations of exogenously administrated BV. We used miRFP703 as an additional reference since it shares the highest amino acid identity (~93%) with miRFP2 and was reported to have higher‐than‐miRFP2 molecular brightness^20^, ^36^ (Table 2). Addition of BV at 62.5 μM resulted in 3.2‐ and 3.7‐fold increases in intracellular brightness of miRFP and miRFP703 while miRFP2 showed only a 1.8‐fold increase (Supplementary Figure 8). The data suggested that miRFP2 has higher BV binding affinity and as a result a larger fraction of miRFP2 exists in the BV‐bound state in HEK cells, which potentially can account for its higher intracellular brightness over miRFP and miRFP703. Overall, characterization in vitro and in cell culture showed that enhancement of intracellular brightness was not due to changes in spectral properties or oligomeric state of the proteins but rather due to improving either molecular brightness in case of TagRFP658 or cofactor binding affinity in case of miRFP2.

**FIGURE 3:**
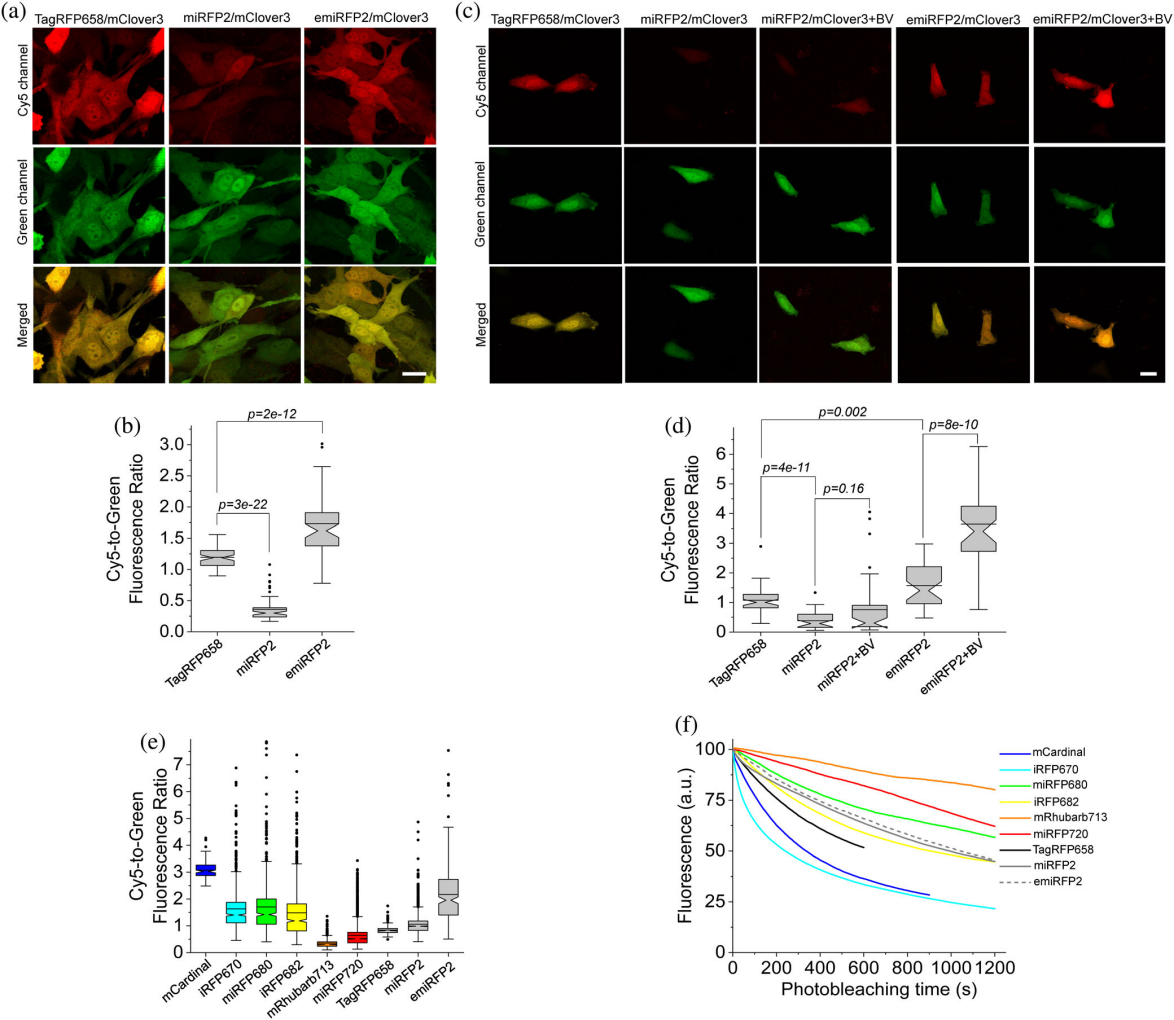
Fluorescence imaging of TagRFP658, miRFP2, and emiRFP2 expressed in mammalian and zebrafish cell cultures. (a) Representative fluorescence images of live NIH3T3 mouse embryonic fibroblasts co‐expressing TagRFP658, miRFP2, and emiRFP2 with mClover3 under the EF1α:2xCMV:EF1α bidirectional promoter (*n* = 67, 62, and 59 cells, respectively, from one transfection each; the dynamic range of the images for the same channel was kept constant throughout the Panels (b) and (d)). Imaging conditions: Cy5 channel, excitation 633 nm from a laser, emission 660–785 nm; green channel, excitation 488 nm from a laser, emission 495–530 nm). (b) Cy5‐to‐green fluorescence ratio of live NIH3T3 fibroblasts shown in (a) (*n* = 67, 62, and 59 cells for TagRFP658, miRFP2, and emiRFP2, respectively, from one transfection each; Kruskal–Wallis analysis of variance [ANOVA]). Box plots with notches are used in this figure (see Figure 1c for the description). (c) Representative fluorescence images of live Pac2 zebrafish embryonic fibroblasts co‐expressing TagRFP658, miRFP2, and emiRFP2 with mClover3 under the EF1α:2xCMV:EF1α bidirectional promoter with and without addition of 25 μM BV for 3 hr before imaging (*n* = 49, 36, 41, 39, and 39 cells for TagRFP658, miRFP2, miRFP2 + BV, emiRFP2, and emiRFP2 + BV, respectively, from one transfection each). Imaging conditions as in (a). (d) Cy5‐to‐green fluorescence ratio of live Pac2 fibroblasts shown in (c) with and without addition of 25 μM BV for 3 hr before imaging (*n* = 49, 36, 41, 39, and 39 cells for TagRFP658, miRFP2, miRFP2 + BV, emiRFP2, and emiRFP2 + BV, respectively, from one transfection each; Kruskal–Wallis ANOVA). Imaging conditions as in (a). Scale bars, 20 μm. (e) Cy5‐to‐green fluorescence ratio for near‐infrared‐fluorescent proteins (NIR‐FPs) co‐expressed with GFP via the P2A peptide in live HEK cells (*n* = 144; 775; 612; 837; 446; 2,937; 226; 1,981; and 271 cells for mCardinal, iRFP670, miRFP680, iRFP682, mRhubarb713, miRFP720, TagRFP658, miRFP2, and emiRFP2, respectively, from two independent transfections each). Box plots with notches are used in this figure (see Figure 1c for the full description). Imaging conditions: Cy5 fluorescence, excitation 635/22 nm from 637 nm laser, emission 730/140 nm; green fluorescence, excitation 478/24 nm for an LED; emission 535/46 nm. (f) Photobleaching curves for NIR‐FPs expressed in live HEK cells under continuous wide‐field illumination from 637 nm laser at 66 mW/mm^2^ (*n* = 55, 32, 10, 43, 32, 48, 56, 38, and 37 cells for mCardinal, iRFP670, miRFP680, iRFP682, mRhubarb713, miRFP720, TagRFP658, miRFP2, and emiRFP2, respectively, from two independent transfections each)

Next, we compared intracellular brightness of TagRFP658 and miRFP2 in NIH3T3 mouse embryonic fibroblasts and PAC2 zebrafish embryonic fibroblasts under identical imaging conditions. While finalizing this study, it was reported that swapping the N‐terminus of the *Rp*BphP1‐derived miRFPs with that of the *Rp*BphP2 protein can significantly improve intracellular brightness in mammalian cells without affecting molecular brightness.^20^ Following the described strategy, we generated an enhanced version of miRFP2, or emiRFP2 for short, and used it for side‐by‐side comparison with TagRFP658 and miRFP2. To perform expression‐level independent quantification of intracellular brightness, the NIR FPs were co‐expressed with mClover3 under the EF1α:2xCMV:EF1α bidirectional promoter (Figure 3a,c). Under transient expression in NIH3T3 cells, emiRFP2 exhibited 1.4‐ and 4.8‐fold higher fluorescence than TagRFP658 and miRFP2, respectively, when quantified using Cy5‐to‐green fluorescence ratio (Figure 3b; here and below we reported fluorescence intensities without normalization to excitation and emission efficiencies for spectrally distinct FPs, unless otherwise indicated, to provide direct comparison of intracellular brightness in real experimental settings rather than intrinsic properties of the FPs). Similar results were obtained in PAC2 fibroblasts with emiRFP2 being 1.5‐ and 4.1‐fold brighter than TagRFP658 and miRFP2, respectively (Figure 3d). Administration of exogenous BV at 25 μM in PAC2 cells further increased fluorescence of miRFP2 and emiRFP2 by 2‐ and 2.3‐fold, respectively (Figure 3d). Consistently with the previously reported results,^20^ swapping the N‐terminus of miRFP2 significantly improved its intracellular brightness. The emiRFP2 protein also outperformed TagRFP658 in terms of intracellular brightness in Cy5 channel.

It is important to validate if the FPs optimized in mammalian cells outperform their counterparts evolved in a bacterial system. Therefore, we decided to compare intracellular brightness and photostability of TagRFP658, miRFP2, and emiRFP2 to spectrally similar GFP‐like and BphP‐based NIR‐FPs in HEK cells using a standard Cy5 filter set with a wide band‐pass emission filter (730/140 nm), which allows efficient collection of NIR fluorescence. Based on the literature search, we selected a set of monomeric and dimeric NIR FPs including mCardinal,^11^ iRFP670,^37^ miRFP680,^20^ iRFP682,^37^ mRhubarb713,^38^ and miRFP720,^39^ which were reported to have high performance in cultured mammalian cells. To account for the expression level during transient transfection of HEK cells, the NIR FPs were co‐expressed with EGFP using the self‐cleavage P2A peptide.^40^ The intracellular brightness of emiRFP2 was higher than all other assessment BphP‐based NIR FPs, but about 1.4‐fold lower than mCardinal (Figure 3e, see Table 2 for the quantification of all proteins). It should be noted that the expression level of the FPs as assessed by fluorescence intensity of GFP was comparable for all expressed constructs. Intracellular photostability of TagRFP658, miRFP2, and emiRFP2 measured under continuous wide‐field illumination was ~2, 3, and 3‐fold higher than that of mCardinal, respectively, however, lower than that for miRFP680, mRhubard, and miRFP720 (Figure 3f and Table 2). Based on the assessed characteristics, emiRFP2 and miRFP680 exhibited the best combination of brightness and photostability among the tested NIR FPs. However, as for the majority of in vivo applications, intracellular brightness is usually the most crucial property, we decided to use mCardinal as a major reference for further characterization of the developed NIR FPs in neurons. Moreover, mCardinal outperformed other recently published NIR FPs, such as mMaroon,^41^ and miRFP670nano,^19^ when compared side‐by‐side in HEK cells (Supplementary Figure 9).

### 2.3 Characterization of TagRFP658 and (e)miRFP2 in neurons

**FIGURE 4:**
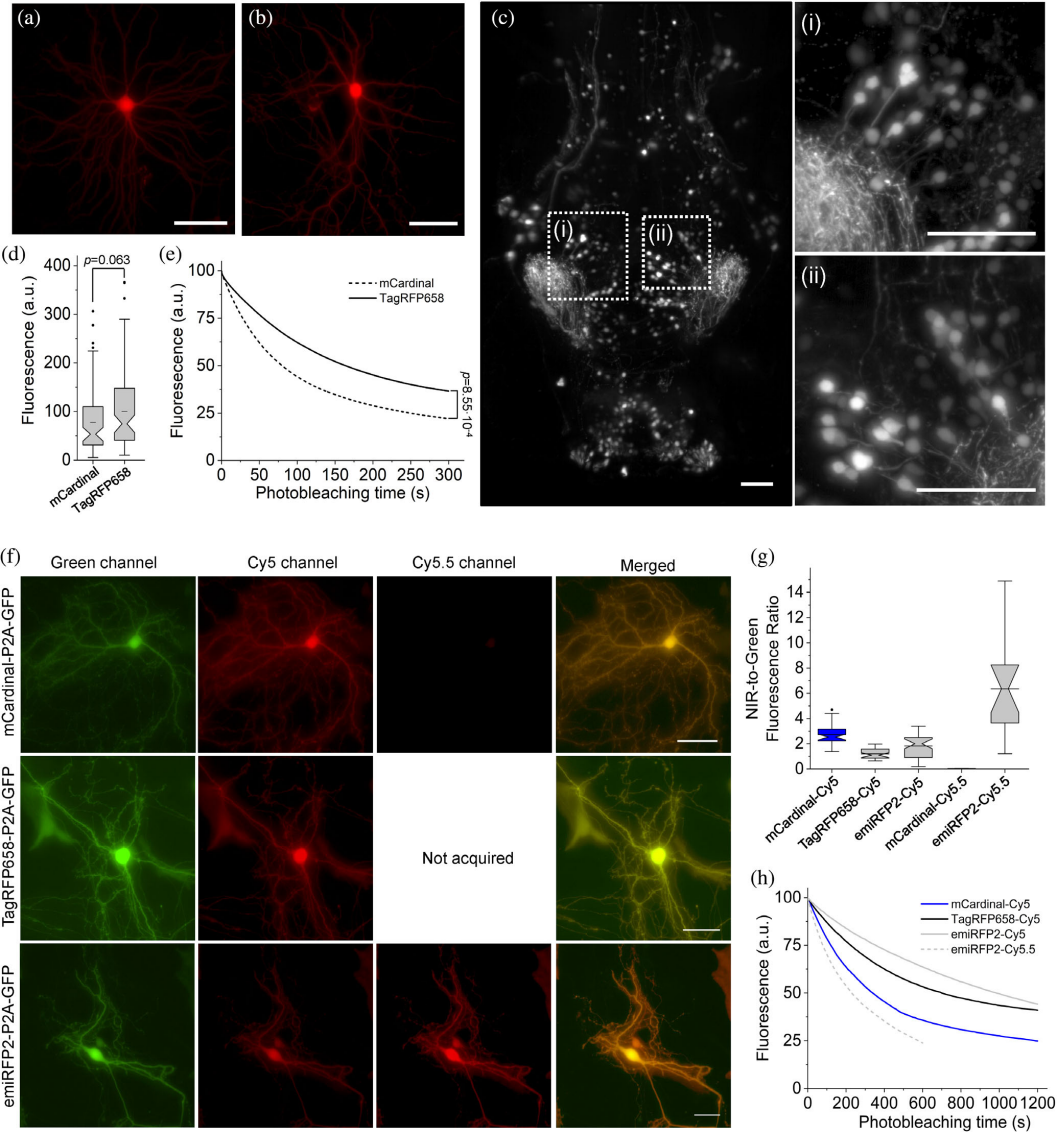
Intracellular brightness and photostability of mCardinal, TagRFP658, and emiRFP2 in live cultured hippocampal mouse neurons and in vivo in zebrafish larvae. (a,b) Representative fluorescence images of primary cultured mouse hippocampal neurons expressing TagRFP658 at (a) 14 and (b) 23 days in vitro (DIV; *n* = 53 and 33 neurons, respectively, from two independent cultures). Imaging condition: excitation 631/28 nm from an LED, emission 664LP. (c) Representative light sheet image of head of zebrafish larvae at 4 days postfertilization expressing TagRFP658 in neurons (*n* = 10 fish from two independent injections). Imaging conditions: excitation 638 nm from a laser, emission 665LP. (i, ii) High‐magnification images of the respective regions shown in white boxes in e. Scale bars, 50 μm. (d) Relative fluorescence of cultured mouse hippocampal neurons expressing mCardinal and TagRFP658 (*n* = 78 and 85 neurons, respectively, from two independent cultures for each protein; one‐way analysis of variance [ANOVA]). Imaging conditions as in (a). Box plots with notches are used in this figure (see Figure 1c for the full description). (e) Raw photobleaching curves for mCardinal (dashed line) and TagRFP658 (solid line) in primary cultured mouse hippocampal neurons (*n* = 9 and 7 neurons, respectively, from one culture each; one‐way ANOVA). Imaging condition: excitation 631/28 nm from an LED at 70 mW/mm^2^, emission 664LP. (f) Representative fluorescence images of cells transfected with pAAV‐CAG‐mCardinal‐P2A‐GFP (top), pAAV‐TagRFP658‐P2A‐GFP (middle), and pAAV‐emiRFP2‐P2A‐GFP (*n* = 39, 33, and 41 neurons from three, two, and three independent transfections from one culture each for mCardinal, TagRFP658, and emiRFP2, respectively, for Cy5 channel and *n* = 15 and neurons from one independent transfection from one culture each for mCardinal and emiRFP2, respectively, for Cy5.5 channel). Imaging conditions: Cy5 channel: excitation 635/22 nm from 637 nm laser, emission 730/140 nm; Cy5.5 channel: excitation 680/13 nm from 680 nm laser, emission 710 LP; GFP channel: excitation 478/24 nm for an LED; emission 535/46 nm. Images in Cy5 and Cy5.5 were taken under matching excitation intensity (66 mW/mm^2^) and the same exposure time (100 ms). The dynamic range of fluorescence intensity in Cy5 and Cy5.5 channels are identical across all images. Scale bar, 20 μm. (g) Near‐infrared (NIR)‐to‐green fluorescence ratio for mCardinal, TagRFP658, and emiRFP2 for the experiment shown in (f). (h) Intracellular photostability of mCardinal, TagRFP658, and emiRFP2 in Cy5 and Cy5.5 channels (*n* = 8, 7, and 9 neurons from three, two, and three independent transfections from one culture each for mCardinal, TagRFP658, and emiRFP2, respectively, under Cy5 excitation and *n* = 5 neurons from one transfection from one culture for emiRFP2 under Cy5.5 excitation). Imaging conditions the same as in (a)

We evaluated the utilization of TagRFP658, miRFP2 and emiRFP2 as NIR fluorescent probes for neuronal labeling under one photon microscopy using standard Cy5 and Cy5.5 filter sets. As a reference, we chose to use mCardinal due to its superior intracellular brightness compared to all other tested NIR‐FPs (Table 2). First, we expressed TagRFP658 in primary hippocampal mouse neurons and in a subset of neurons in live zebrafish larvae. In both preparations, the fluorescence of TagRFP658 was evenly distributed within the cytosol, individual dendrites, and nucleus of live neurons without any aggregation or nonspecific localization (Figure 4a–c). In cultured neurons, TagRFP658 was about 28% brighter and twice more photostable than mCardinal (photobleaching half‐time for TagRFP658 and mCardinal was 164 and 80 s, respectively; Figure 4d,e). Using whole‐cell patch clamp recordings, we showed that TagRFP658 expression did not alter membrane resistance, membrane capacitance, or the resting potential of cultured mouse neurons (Supplementary Figure 10).

Next, we assessed performance of emiRFP2 in cultured neurons side‐by‐side with mCardinal and TagRFP658. Since emiRFP2 outperformed miRFP2 in earlier experiments it was not used for quantitative imaging in neurons, although miRFP2 can be efficiently expressed and imaged in cultured neurons both under transient transfection and rAAV transduction (Supplementary Figure 11). The fluorescence of the NIR FPs co‐expressed with EGFP via P2A peptide was evenly distributed within the cytosol, individual dendrites, and nucleus of live cultured neurons without any aggregation or nonspecific localization (Figure 4f). Quantification of fluorescence intensity revealed that emiRFP2 were about 3.5‐times brighter in Cy5.5 channel than in Cy5 channel, while the mCardinal fluorescence in Cy5.5 channel was almost undetectable (Figure 4g). When quantified by Cy5‐to‐green fluorescence ratio, mCardinal was 2.2‐ and 1.5‐fold brighter than TagRFP658 and emiRFP2, respectively. However, when raw mean fluorescence values in Cy5 channel were compared, TagRFP658 was about 20% brighter than mCardinal, similarly to the results shown in Figure 4c (note comparable fluorescence intensity for the representative images of mCardinal and TagRFP658 in Cy5 channel, but significantly dimmer GFP fluorescence in case of mCardinal‐P2A‐GFP construct; Supplementary Figure 12). Intracellular photostability of emiRFP2 was four‐fold lower under Cy5.5 illumination compared to Cy5 excitation (photobleaching half‐times were 230 and 990 s under Cy5.5 and Cy5 illumination, respectively; Figure 4h). At the same time, emiRFP2 exhibited superior intracellular photostability compared to mCardinal and TagRFP658 in the Cy5 channel, which were characterized by photobleaching halftime of 340 and 700 s, respectively, closely matching corresponding values obtained in HEK cells (Figure 4h and Table 2). Similar correlations of intracellular brightness and photostability in Cy5 and Cy5.5 channels were also observed in live HEK cells (Supplementary Figure 12). These results demonstrated that Cy5.5 channel provided high efficiency of the emiRFP2 fluorescence imaging; however, the gain in brightness came at the cost of reduced photostability.

**FIGURE 5:**
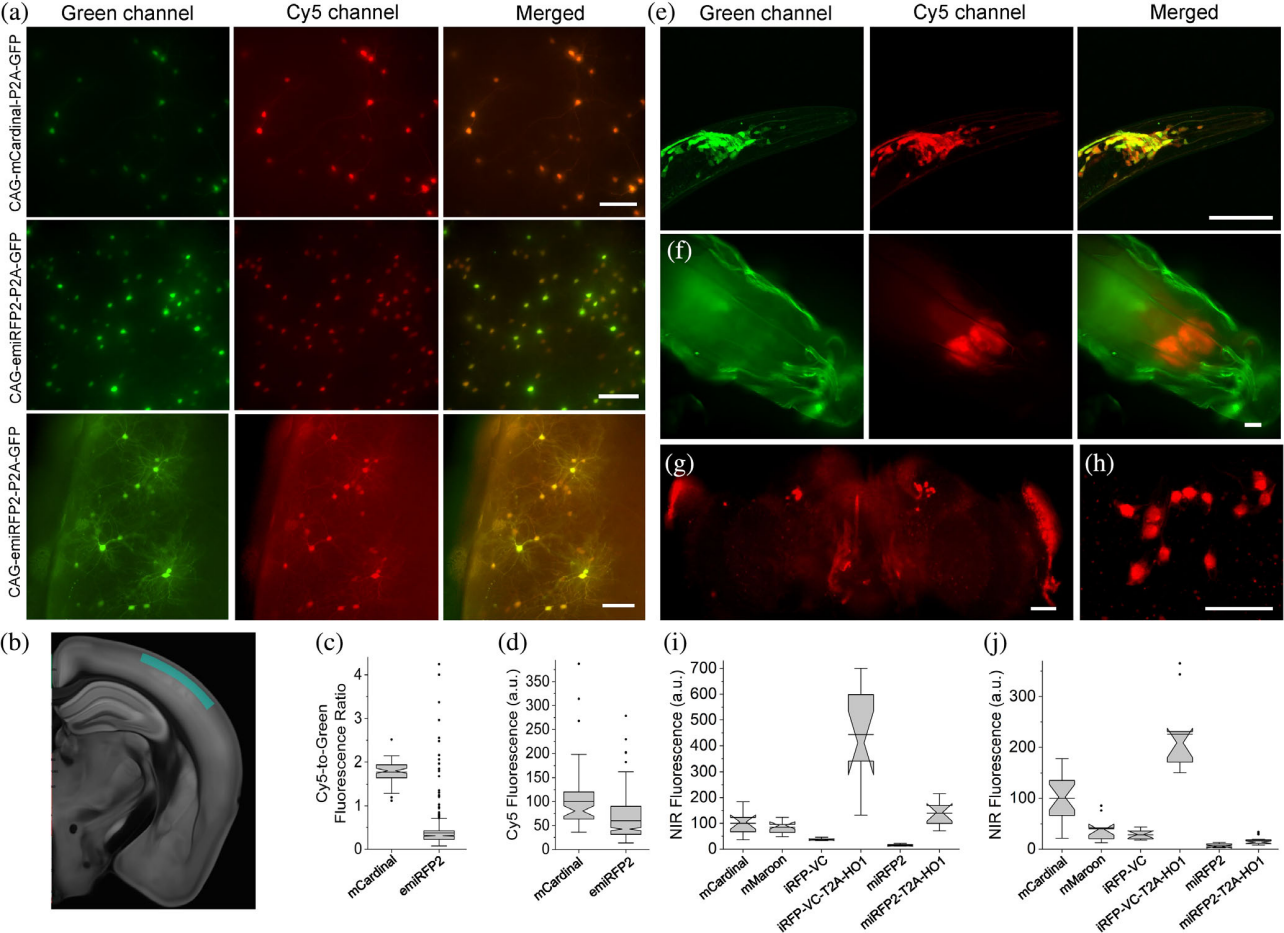
In vivo imaging and characterization of miRFP2 and emiRFP2. (a) Representative fluorescence images of live brain slices expressing mCardinal‐P2A‐GFP (top) and emiRFP2‐P2A‐GFP (middle and bottom; *n* = 6 slices from three mice each; L1 and L2/3 of somatosensory cortex, see Panel (b) for brain map). Imaging conditions: green channel: excitation 478/24 nm for an LED, emission 535/46 nm; Cy5 channel: excitation 635/22 nm from 637 nm laser, emission 730/140 nm. Top and middle rows of images are presented using the same dynamic range to facilitate visual comparison of mCardinal and emiRFP2 expressing neurons, bottom row images have adjusted LUTs to visualize processes of the emiRFP2 positive neurons. Scale bars, 100 μm. (b) Brain section indicating imaged region highlighted in marine blue (image from kimlab.io/brain‐map). (c) Cy5‐to‐green fluorescence ratio of live neurons shown in (a) (*n* = 67 and 803 neurons from three mice each for mCardinal and emiRFP2, respectively). Imaging conditions same as in (a). (d) Cy5 mean fluorescence of live neurons shown in (a) (*n* = 67 and 803 neurons from three mice each for mCardinal and emiRFP2, respectively). Imaging conditions same as in (a). (e) Representative fluorescence images of the *Caenorhabditis elegans* head co‐expressing codon‐optimized genes of miRFP2‐T2A‐HO1 and jGCaMP7b in neurons (*n* = 15 worms from two independent microinjections). Imaging conditions: green channel, excitation 488 nm from a laser, emission 500–650 nm; Cy5 channel, excitation 631 nm from a laser, emission 645–700 nm. Scale bar, 50 μm. (f) Representative fluorescence images of live intact third instar *Drosophila* larva expressing miRFP2‐T2A‐HO1 (*n* = 10 larvae from two transgenic lines). Imaging conditions: green channel, excitation 475/34 nm from LED, emission 527/50 nm (green fluorescence corresponds to autofluorescence); Cy5 channel, excitation 631/28 nm from LED, emission 665LP. Scale bar, 100 μm. (g) Representative low‐magnification fluorescence image of brain explant from adult *Drosophila* fly expressing codon‐optimized gene of miRFP2‐T2A‐HO1 in R84C10‐GAL4 line (*n* = 5 brains from one transgenic lines). Imaging conditions: excitation 635/22 nm from 637 nm laser, emission 665LP. Scale bar, 50 μm. (h) Representative high‐magnification fluorescence image of brain explant from adult *Drosophila* fly expressing codon‐optimized miRFP2‐T2A‐HO1 in R84C10‐GAL4 line (*n* = 5 brains from one transgenic lines). Imaging conditions: excitation 631 nm from a laser, emission 645–700 nm. Scale bar, 50 μm. (i) Relative near‐infrared (NIR) fluorescence of mCardinal, mMaroon, iRFP‐VC, iRFP‐VC‐T2A‐HO1, miRFP2, and miRFP2‐T2A‐HO1 expressed pan‐neuronally in *Drosophila* third instar larvae (*n* = 10, 12, 7, 11, 9, and 10 larvae from one transgenic line each, respectively). Imaging conditions same as in (h). (j) Relative Cy5 fluorescence of mCardinal, mMaroon, iRFP‐VC, iRFP‐VC‐T2A‐HO1, miRFP2, and miRFP2‐T2A‐HO1 expressed pan‐neuronally in adult *Drosophila* fly (*n* = 19, 10, 11, 11, 11, and 21 flies from one transgenic line each, respectively). Imaging conditions same as in (h)

High performance of miRFP2 and emiRFP2 in cultured neurons encouraged us to express them in vivo in model organisms such as mice, *C. elegans*, and *Drosophila melanogaster*. First, we co‐expressed mCardinal and emiRFP2 with GFP in cortical neurons in mice via in utero *electroporation* and performed imaging of the expressed proteins in acute brain slices at around P28. The emiRFP2 protein expressed in vivo showed even distribution in cell bodies and processes without aggregation (Figure 5a,b). Quantitative imaging showed that mCardinal had 4.8‐fold higher Cy5‐to‐green fluorescence ratio than emiRFP2; however, mean fluorescence intensity of mCardinal was only 1.7‐fold higher than that of emiRFP2 (Figure 5c,d; note significantly lower green fluorescence intensity in the representative image for CAG‐mCardinal‐P2A‐GFP construct compared to CAG‐emiRFP2‐P2A‐GFP in Figure 5a). In addition, values for Cy5‐to‐green fluorescence ratio in the case of emiRFP2 exhibited significant variability ranging from 0.07 to 4.2 versus only 1.1 to 2.5 for mCardinal. Next, we expressed the codon‐optimized genes of miRFP2 using pan‐neuronal expression systems in transgenic *C. elegans* and in *Drosophila* fruit flies. In case of *C. elegans*, we did not observe any specific NIR signal, while NIR fluorescence in larvae and adult fruit flies was clearly detectable although its intensity was several times lower than in cultured neurons under the same imaging conditions (Supplementary Figure 13). Reduced fluorescence of miRFP2 can be due to the insufficient concentration of the BV cofactor in worms and flies. Previously, it was shown that co‐expression of heme oxygenase‐1 (HO1) in worms and flies can enable fluorescence of the BphP‐based FPs^42^ and biosensors.^43^ To optimize conditions for miRFP2 expression, we constructed two bicistronic vectors using IRES2 and a viral 2A cleavage sequence to co‐express miRFP2 and HO1 and transfected them into HEK cells. Quantification of NIR fluorescence in HEK cells revealed that HO1 co‐expression via IRES2 and P2A improves miRFP2 brightness by 40 and 83%, respectively (Supplementary Figure 14). Therefore, we decided to use the viral P2A cleavage sequence to co‐express codon‐optimized genes of miRFP2 and HO1 in neurons in worms and flies. Indeed, co‐expression of HO1 enabled bright NIR fluorescence of miRFP2 in *C. elegans* and in larvae and adult fruit flies allowing visualization of individual neurons (Figure 5e–h). To validate miRFP2 performance in fruit flies we decided to compare its fluorescence to that of other GFP‐like and BphP‐derived NIR FPs under identical expression conditions. We establish transgenic lines with pan‐neuronal expression of *Drosophila* codon optimized genes of mCardinal, mMaroon, iRFP‐VC (aka iRFP713/V256C), and iRFP‐VC‐P2A‐HO1 and quantified their Cy5 fluorescence in live intact third instar larva and adult fruit flies (Figure 5i,j). Co‐expression of HO1 significantly enhanced brightness of miRFP2 and iRFP‐VC both in adult flies and in larvae, although increase of miRFP2 brightness in adult flies was less pronounced than that in larva, 2.6‐fold versus 9‐fold. In the case of iRFP‐VC, fluorescence enhancement in larva and adult flies was comparable, about 12‐ and 8‐fold, respectively. While miRFP2‐T2A‐HO1 outperformed mCardinal and mMaroon in terms of brightness in third instar larva, it was noticeably dimmer in adult fruit flies (Figure 5i,j). At the same time, co‐expression of iRFP‐VC with HO1 resulted in the brightest fluorescence among the tested proteins both in larva and adult flies. Thus, we demonstrated that miRFP2 is a suitable NIR FP for imaging neurons in culture and in vivo in small model organisms, like worms and flies, and outperformed other high performing NIR FPs under certain conditions, for example, in fruit fly larva. Our data also showed that co‐expression of HO1 in worms and flies are essential for achieving sufficient brightness of BphP‐based NIR FPs in *C. elegans* and *Drosophila* flies.

### 2.4 Multicolor imaging in cell culture and in vivo

**FIGURE 6:**
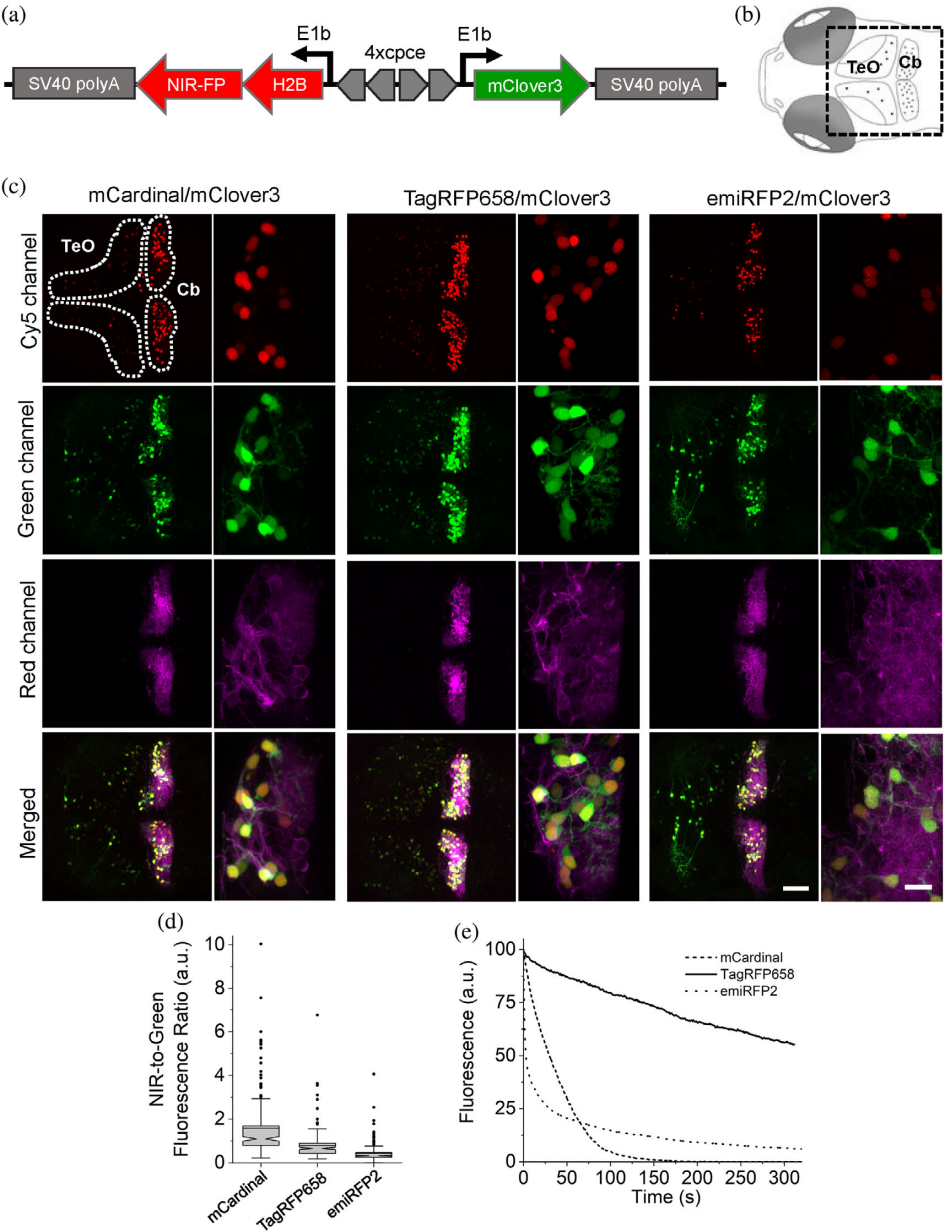
Three‐color in vivo imaging of zebrafish larvae. (a) Schematic drawing of a bidirectional PC‐specific expression system using 4x PC specific regulatory element (cpce) to co‐express near‐infrared (NIR)‐FP‐H2B fusion protein with mClover3. (b) Schematic drawings of dorsal view of 4 days postfertilization (dpf) larval zebrafish brain region delineating the optic tectum (TeO) and the cerebellum (Cb). The region of interest enclosed by a square was recorded in images shown below. (c) Representative confocal images of 4 dpf zebrafish larvae transiently co‐expressing H2B‐NIR‐FP with mClover3 (expression of this construct occurs in a mosaic manner) in stable transgenic background with PC specific membrane targeted mScarlet. The TeO and the cerebellar region (Cb) are enclosed by the white dashed lines. Each subset of images (left, whole overview of tecum and cerebellar region; right, higher magnified images depicting PCs) shows the expression of each H2B‐NIR‐FP fusion (left, mCardinal; middle, TagRFP658; right, emiRFP2), cytoplasmic mClover3, membrane targeted mScarlet, and the overall merged image (upper to lower; *n* = 4 fish for each subset). Imaging conditions, NIR channel: excitation 633 nm laser, emission 722/63 nm; green channel: excitation 488 nm laser, emission 513/17 nm; red channel: excitation 561 nm laser, emission 585/15 nm. Scale bars: 50 μm (overviewed images), 10 μm (higher magnified images) (d) NIR‐to‐green fluorescence ratio for NIR‐FP expressing PCs (*n* = 175, 161, 192 cells for mCardinal, TagRFP658, and emiRFP2, respectively, from four fish each; imaging conditions as in (c)). Box plots with notches are used (see caption of Figure 1c for the full description). (e) Photostability curves for mCardinal (dashed line), TagRFP658 (solid line), and emiRFP (dotted line) expressed in PCs (*n* = 4 cells for each from one fish each)

We tested the feasibility of TagRFP658 and miRFP2 in multicolor neuronal imaging in combination with green and red FPs expressed in larval zebrafish cerebellar Purkinje cells (PCs). First, we generated a set of the bidirectional expression constructs (Figure 6a) using zebrafish codon‐optimized genes of NIR FPs allowing to co‐express a nuclear‐localized H2B histone fused to either mCardinal, TagRFP658, miRFP2, or emiRFP2 with cytoplasmic green FP mClover3 and transfected them into NIH3T3 cells stably expressing trans‐Golgi network protein 46 (TGN46) fused to red FP mScarlet. Imaging using standard filter configurations under confocal microscope allowed for crosstalk‐free triple color imaging for each combination of the selected FPs (Supplementary Figure 15). Next, we expressed the selected FPs in specific neuronal subpopulations of zebrafish larvae, especially targeting cerebellar PCs using the same constructs but carrying a PC‐specific bidirectional promoter^44^ instead of the ubiquitous CMV‐EF1 promoter. As the enhanced variant of miRFP2, emiRFP2, exhibited higher intracellular brightness both in NIH3T3 cells and zebrafish PAC2 fibroblast than that of the original miRFP2 (Figure 3b,d), it was selected for expression in zebrafish larva. Using a standard confocal microscope, we easily visualized NIR fluorescence in PC nucleus using Cy5 channel together with mClover3 distributed throughout entire PC's cytoplasm using green channel, while imaging in red channel provided clear visualization of the PCs axonal and/or dendritic structures with membrane tagged mScarlet fluorescence whose expressions are predominantly detected in PCs together with slight expression in tectal neurons (Figure 6b,c). Thus, NIR fluorescence of the tested NIR FPs were easily distinguishable from mScarlet fluorescence and thus can be used to label multiple neuronal compartments in conjunction with additional blue, or green FPs. We further quantified the brightness and photostability of mCardinal, TagRFP658, and emiRFP2 fluorescence by expressing corresponding constructs in PCs of less pigmented brass embryos (Supplementary Figure 16). To account for expression level of the FPs, we calculated NIR‐to‐green fluorescence ratio for single PCs in zebrafish embryos. Fluorescence quantification revealed that mCardinal fluorescence was about twice higher than that of TagRFP658, whereas emiRFP2 exhibited 3.6‐fold lower brightness than mCardinal (Figure 6d). Photobleaching experiments performed under identical excitation power for all selected FPs demonstrated 10‐times higher photostability of TagRFP658 over mCardinal. However, emiRFP2 fluorescence exhibited rapid decay with half‐time about 3 s making emiRFP2 about 10‐fold less photostable than mCardinal at least in this zebrafish model (Figure 6e). Despite the lower intracellular brightness of TagRFP658 than mCardinal, it can be a more practical fluorescent tag for live imaging due to significantly higher photostability.

**FIGURE 7:**
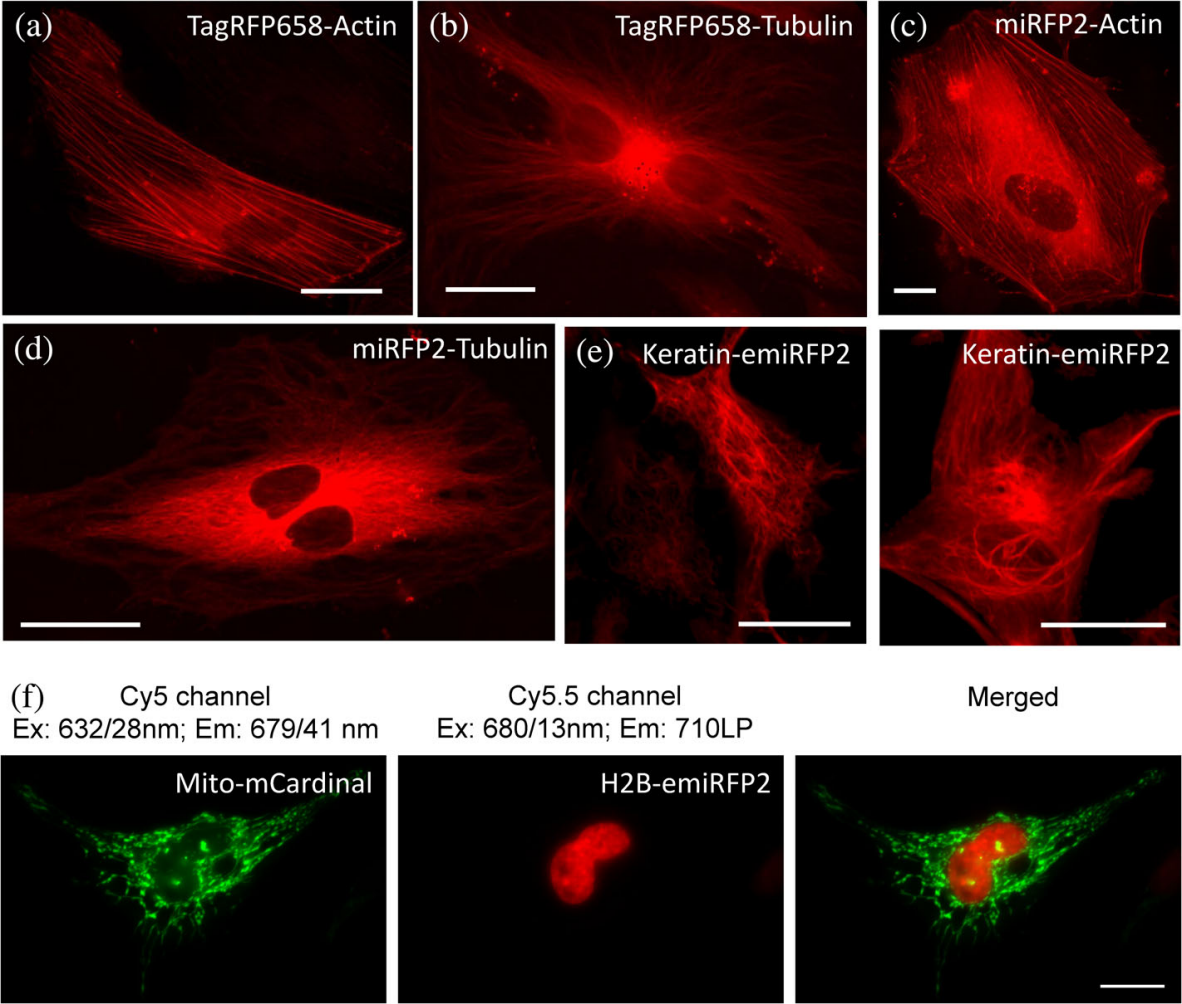
Fluorescence imaging of TagRFP658, miRFP2, and emiRFP2 fusions expressed in HeLa cells. (a–e) Representative fluorescence images of live HeLa cells transfected with (a) TagRFP658‐α‐Tubulin (*n* = 11 cells from two independent transfections) and (b) TagRFP658‐β‐actin (*n* = 19 cells from two independent transfections). (c) miRFP2‐α‐Tubulin (*n* = 15 cells from two independent transfections), (d) miRFP2‐β‐actin (*n* = 21 cells from two independent transfections), and (e) Keratin‐emiRFP2 (*n* = 8 cells from two independent transfections). Imaging conditions: (a–d) excitation 631/28 nm from an LED, emission 664LP under wide‐field microscope. (e) Excitation 640 nm from laser, emission 670–750 nm under confocal microscope. (f) Dual‐color near‐infrared (NIR) imaging of live HeLa cells co‐expressing Mito‐mCardinal and H2B‐emiRFP2 (*n* = 12 cells from two independent transfection)

Earlier we demonstrated that (e)miRFP2 imaging in Cy5.5 channel is several times more efficient than in Cy5 channel, while mCardinal fluorescence is not detectable in Cy5.5 channel (Figure 4f,g and Supplementary Figure 12). Therefore, we decided to explore a possibility for dual‐color NIR imaging of subcellular structures using combination blue‐shifted and red‐shifted NIR FPs, for example, mCardinal and emiRFP2. First, we verified that the developed NIR FPs can be properly localized in fusions with structured proteins in mammalian cells. Indeed, fusions of TagRFP658 and (e)miRFP2 with α‐tubulin, β‐actin, and keratin demonstrated proper localization in cultured mammalian cells (Figure 7a–f). Next, we co‐expressed Mito‐mCardinal and H2B‐emiRFP2 fusions in HeLa cells and acquired images in Cy5 and Cy5.5 channels. To further improve spectral separation, we swapped the wide band‐pass emission filter in our standard Cy5 channel with a narrower emission filter 679/41 nm. This optical setup enabled crosstalk‐free imaging of mCardinal and emiRFP2 (Figure 7g). The blue‐shifted and red‐shifted NIR FPs can further increase spectral multiplexing of fluorescence imaging in combination with other common FPs.

## 3 DISCUSSION

Here, we described a practical and simple approach for rapid optimization of FPs via directed molecular evolution in mammalian cells. The presented method does not require generation of stable cell lines or viral vector preparation and can be carried out using commonly available high‐throughput cell sorting hardware such as FACS. The simplicity and accessibility of the presented method are the major advantages compared to previously described methods, which either involve the generation of stable lines^29^, ^45^ or require custom‐built and/or expensive hardware such as high precision DMDs,^46^ microfluidic sorters,^47^ and microraft arrays.^48^ Furthermore, the present method is also faster than alternative ones. One iteration of directed evolution accomplished with selecting individual variants can be performed in a course of ~8 days, which is on average five to eight times faster than alternative methods involving stable cell line generation^26^, ^28^, ^29^ or viral vector production.^27^, ^47^, ^49^ The faster timeline compared to previously reported strategies was mainly achieved due to the utilization of transient transfection of large gene libraries in combination with fast target gene recovery from small pools of collected cells (~50–100 cells). Besides a faster timeline, transient transfection provides additional advantages over traditional methods of gene library expression. It enables the evolution of proteins for which it is impossible or very hard to establish stable cell lines, such as, for example, opsins.^33^ In addition, it is more accessible than other methods as it only requires very standard expression vectors containing SV40*ori*, widely used HEK293T cells, and cheap transfection reagents. Besides clear advantages, there are also other experimental considerations that should be taken into account. Conditions of gene delivery are optimized so that most of the transfected cells (~50%) receive a single gene copy at total transfection efficiency of about ~5%, which is similar to lentiviral transduction at a multiplicity of infection of ~0.1 that also used single gene copy delivery.^27^, ^50^, ^51^ Therefore, in the case of large libraries, it may significantly extend screening time as 95% of cells are negative. To reduce screening time using instrumental methods, negative cells can be removed using selective markers of choice, which can be included in the expression vector, although it will extend the timeline as antibiotic selection usually takes 3–4 days or so. Furthermore, about 50% of transfected cells contain more than one gene,^33^ which correspondingly requires screening of about twice more individual clones (Steps 7–9 of workflow in Figure 1a) than cells that were collected during screening in order to ensure coverage of all diversity of recovered genes. However, it should be noted that lentiviral vectors provide similar efficiency of single gene copy delivery as gene delivery via calcium phosphate transfection, and viral transduction is a stochastic process with the same probability distribution. The developed method was tested only in HEK293T cells because this cell line provides sufficient expression level upon single‐copy plasmid transfection due to episomal replication of DNA facilitated by the SV40*ori* sequence in the expression vector. Besides HEK293T, there are several other commercially available cell lines expressing the large T antigen required for episomal replication (available via ATCC) as well as self‐replicating plasmids,^52^ which can provide a high enough expression level under required transfection conditions. However, applicability of other cell lines and expression vectors remains to be tested and verified. In this study, we utilized FACS for high‐throughput screening of mammalian cells, however, any other method for fluorescence cell screening can be integrated into the described workflow. For example, the SPOTlight approach,^46^ microfluidic sorter,^47^ photostick technique,^53^ or robotic cell picker^33^ can be implemented following FACS enrichment of transfected cells, thus enabling phenotyping of single cells for a diversity of biochemical and photophysical properties of expressed mutated proteins, for example, subcellular localization, pH stability, photostability, quantum yield, and so forth.

The developed approach was validated by enhancing intracellular brightness of green and NIR FPs derived from evolutionary distinct naturally occurring chromoproteins. The starting template proteins, phiLOV2.1, TagRFP657, and miRFP, which were already highly optimized via directed molecular evolution either in *E. coli* or in mammalian cells, were significantly enhanced just in two iterations of directed evolution. We demonstrated that oligomerization or alternation of fluorescence spectra cannot account for the increase in intracellular brightness. In the case of TagRFP657, the enhancement of intracellular brightness can be most likely explained by improvements in chromophore maturation as TagRFP658 is characterized by a higher extinction coefficient compared to its precursor. However, for phiLOV2.1 and miRFP, the increase in intracellular brightness does not correspond to changes in molecular brightness. In contrast to GFP‐like FPs, like TagRFP657, phiLOV2.1 and miRFP incorporate exogenous chromophores available in the cytoplasm of mammalian cells. Therefore, their intracellular brightness, besides of course intrinsic photophysical properties, will depend on efficiency of chromophore incorporation and chromophore availability. We revealed that miRFP2 has a higher affinity toward BV than miRFP (Supplementary Figure 8); however, it is hard to precisely quantify for which fraction of intracellular brightness enhancement the increase in affinity is responsible. The exact molecular basis of intracellular brightness increase observed for miRFP2 and phiLOV3 will require further investigations, which are beyond the scope of the present study. Overall, this study provided additional evidence, aligning with many previous reports, that relative molecular brightness of FPs expressed in *E. coli* does not always match intracellular brightness and the observed difference is particularly pronounced for proteins that require exogenous chromophores. The presented results also justify the need for optimization of FPs in mammalian cells for higher performance in vivo.

To date, only four FPs were reported to be developed via directed molecular evolution in mammalian cells. Three of them are GFP‐like FPs, mPlum,^26^ Kriek,^27^ and mCrispRed,^28^ and one is the BphP‐based NIR FP miRFP.^33^ A major rationale for evolving FPs in mammalian cells is to optimize their performance for in vivo applications; however, only mPlum and miRFP were tested in vivo. Crucially, side‐by‐side comparison of mPlum with other GFP‐like FPs did not reveal any advantages of mPlum for in vivo imaging,^54^, ^55^ while miRFP was not compared to other BphP‐based FPs either in cultured cells or in vivo. Therefore, it is important to demonstrate that the developed directed evolution approach is capable of yielding practical FPs that outperform other FPs developed in *E. coli*. First, assessment of the developed FPs in HEK cells showed miRFP2 has comparable intracellular brightness to that of the best performing BphP‐based NIR FPs reported to date, such iRFP670, miRFP680, and iRFP682. However, it should be noted that iRFP670, miRFP680, and iRFP682 although evolved in *E. coli* were actually selected in HeLa cells.^36^, ^37^ In turn, rationally enhanced version of miRFP2 significantly outperformed other BphP‐based NIR FPs in HEK cells and can be considered as the brightest BphP‐based NIR FP in HEK cells (Table 2). Thus, the presented method enabled development of one of the best performing NIR FPs in HEK cells in the class in just five rounds of directed evolution (three rounds to select miRFP and two rounds to select miRFP2). However, further assessment of miRFP2 in vivo in model organisms reveals its inconsistent performance across various preparations. For example, miRFP2 co‐expressed with HO1 was brighter mCardinal in fruit fly larvae, while dimmer in adult flies (Figure 5i,j). Fluorescence of miRFP2 in *C. elegans* can be enabled only by HO1 co‐expression. The intracellular brightness of emiRFP2 was 1.5‐fold dimmer than mCardinal in cultured neurons, but about 4.8‐fold dimmer in brain tissue (Figures 4g and 5c). Furthermore, emiRFP2 was brighter than TagRFP658 in cultured neurons, while dimmer in zebrafish (Figures 4g and 6d). We also observed significantly greater variation of Cy5‐to‐green fluorescence ratio for miRFP2 expressed in neurons in vivo in mice in comparison to cultured neurons that is most likely due to higher variability of BV concentration in vivo. We suggest that high variability of miRFP2 performance in vivo in model organisms is determined by heme metabolism, which is required for BV production. For example, in wild‐type *C. elegans*, heme utilization is not going via the BV intermediate as in vertebrates,^56^, ^57^ and thus expression of miRFP2 itself did not result in NIR fluorescence. However, we demonstrated that co‐expression of HO1 is sufficient to enable BV production in *C. elegans*. Similarly, HO1 co‐expression in Drosophila boosted fluorescence intensity of BphP‐based NIR FPs expressed in neurons. Overall, these results indicated that high intracellular brightness of BphP‐based NIR FPs in cultured cells may not be retained in vivo in model organisms, which complicates selection of the right NIR FP for in vivo applications and thus may require testing several candidates in real settings.

## 4 MATERIALS AND METHODS

### 4.1 Molecular cloning and mutagenesis

The mammalian codon‐optimized genes of phiLOV2.1 and UnaG were synthesized de novo by GenScript based on the amino acid sequences reported in the original publications.^4^, ^31^ The TagRFP657 and miRFP genes were acquired from Addgene (TagRFP657 plasmid#31959; miRFP plasmid#108409). Synthetic DNA oligonucleotides used for cloning were purchased from Integrated DNA Technologies. PrimeStar Max master mix (Clontech) was used for high‐fidelity PCR amplifications. Restriction endonucleases were purchased from New England BioLabs and used according to the manufacturer's protocols. Ligations were performed using T4 DNA ligase (Fermentas) or InFusion HD kits (Clontech). Small‐scale isolation of plasmid DNA was performed with Mini‐Prep kits (Qiagen); large‐scale DNA plasmid purification was done with GenElute HP Endotoxin‐Free Plasmid Maxiprep Kits (Sigma‐Aldrich). Random libraries of mutants were prepared using Mutazyme II DNA polymerase (Agilent) under high mutation rate conditions (9–16 mutations per kilobase pair) and subcloned into the pN1 vector (Clontech). Obtained gene libraries in expression vectors were electroporated into NEB10‐β *E. coli* host cells (New England BioLabs). Serial dilutions (10^−4^ and 10^−5^) of the electroporated cells were plated on LB/agar medium supplemented with 100 mg ml^−1^ kanamycin to estimate electroporation and cloning efficiency. For each library, 20 randomly selected clones were sequenced to confirm ligation efficiency, that is, fraction of the clones containing target genes, and mutation rate. The remainder of the cells was grown overnight in LB medium supplemented with 100 mg ml^−1^ of kanamycin for subsequent plasmid DNA isolation. Library transfection into HEK293FT cells (Invitrogen) was performed as described previously. Briefly, HEK293FT cells were maintained between 10 and 70% confluence at 37°C with 5% CO2 in DMEM medium (Cellgro) supplemented with 10% heat‐inactivated FBS (Corning), 1% penicillin/streptomycin (Cellgro), and 1% sodium pyruvate (BioWhittaker). Transfection of HEK293FT cells with gene libraries was performed using a commercially available calcium phosphate (CaPhos) transfection kit (LifeTechnologies) according to the modified protocol using the pUC19 plasmid as “dummy” DNA in weight ratio library DNA: pUC19 1:100. To sort the gene library‐transfected HEK293FT cells using flow cytometry, cells were harvested from a culture dish ~48 hr after gene library transfection by applying trypsin for 5–10 min (Cellgro) and then washed twice by centrifuging the cell suspension for 5 min at 500 rpm and resuspending cells in PBS (Cellgro). The washed cells were then resuspended in PBS supplemented with 4% FBS (Corning) and 10 mM EDTA at a density of 1–2·10^6^ cells/ml and filtered through a 30‐μm filter (Falcon) to prevent clogging on the FACS machine. The filtered cells were sorted by FACSAria (BD Biosciences) running BD FACS Diva 8.0 software and equipped with standard 488‐ and 640‐nm solid‐state lasers. Debris, dead cells, and cell aggregates were gated out using forward and side scatter before desired fluorescence signals were detected. For each library, several hundred cells exhibiting highest fluorescent intensity in the corresponding channel were collected and subjected to whole genome amplification (WGA) using a commercially available WGA kit (New England BioLabs) followed by PCR amplification. Amplicons with a size corresponding to that of the target gene were purified by agarose gel electrophoresis and cloned into the pN1 vector. Obtained colonies were individually picked to confirm the correct insert using PCR with a pair of primers that anneal to CMV promoter and the target genes. Colonies with correct insert were cultured in LB with 100 mg ml^−1^ of kanamycin for plasmid purification. Purified plasmids were individually transfected into HEK293FT cells using TransIT‐X2 reagent (Mirus). In 24 hr post transfection, HEK cells were imaged using a wide‐field Nikon Eclipse Ti equipped with 10× NA 0.45 and 20× NA 0.75 objective lenses, a SPECTRA‐X light engine (Lumencor) with 475/28 nm, and 631/28 nm exciters (Semrock), a 5.5 Zyla camera (Andor), and automated stage (Ludl), controlled by NIS‐Elements AR software Nikon to assess fluorescence and photostability.

The genes of miRFP720 and miRFP670nano were de novo synthesized by GenScript using sequences reported in the original publications.^19^, ^39^ The genes of mCardinal, mMaroon, and miRFP703 were acquired from Addgene as plasmids #54590, #54554, and #79988, respectively. To construct α‐tubulin and β‐actin fusions, the phiLOV3, TagRFP658, and miRFP2 genes were PCR amplified and swapped with mClover2 in pmClover2‐tubulin‐C‐18 plasmid (Addgene #56376) and with TagRFP675 in pTagRFP675‐actin‐C1 (Addgene #44277) using InFusion cloning (Clontech). To construct Keratin fusions, emiRFP2 were PCR amplified and swapped with miRFP670nano in pKeratin‐miRFP670nano plasmid (Addgene #127437) using Fusion cloning (Takara). To express TagRFP658 and mCardinal in neurons under CaMKII promoter the corresponding genes were PCR amplified and swapped with the Arch‐GFP gene in FCK‐Arch‐GFP (Addgene #22217) using InFusion cloning. The pAAV‐CAG‐miRFP720‐P2A‐GFP and pAAV‐CAG‐miRFP2‐P2A plasmids were constructed by cloning miRFP720‐P2A and miRFP2‐P2A into pAAV‐CAG‐GFP (Addgene #37825) in frame with GFP.

For expression in zebrafish larvae, the genes of mCardinal, TagRFP678, miRFP, and miRFP2 were codon‐optimized using the online resource at http://www.bioinformatics.org/, synthesized de novo by GenScript. The zebrafish codon optimized emiRFP2 gene was cloned by substituting the nucleotides encoding *Rp*BphP1‐based N‐terminus of zebrafish codon optimized miRFP2 (aa 2–19) with those encoding 13 amino acid preceding the chromophore‐binding Cys in *Rp*BphP2 as previously reported.^20^

For co‐expression of TagRFP658, miRFP2, and emiRFP2 with mClover3 in NIH3T3 mouse embryonic fibroblasts (DSMZ) and PAC2 zebrafish embryonic fibroblasts, the zebrafish codon‐optimized genes of the corresponding NIR FPs and mClover3 were cloned into an expression vector carrying the bidirectional promoter (p‐EF1a:2xCMV:EF1a) consisting of two CpG free mCMV enhancers (Invivogen) each followed by CpG free hEF1alpha promoter (Invivogen) and SV40 polyA sequences. The generated expression cassettes were flanked by Tol2 transposon arms. The p‐EF1α:2xCMV:EF1α bidirectional vector allows for expression of two reporter genes with comparable expression levels. For pan‐neuronal expression in zebrafish, the zebrafish codon optimized gene of TagRFP658 was cloned into pTol2‐UAS‐zArchon1‐KGC‐GFP‐ER2 plasmid (Addgene #108427) described before^33^ by swapping zArchon1‐KGC‐GFP‐ER2 using SpeI and AscI sites.

For transient expression in cerebellar Purkinje neurons in zebrafish, EF1a:2xCMV:EF1a promoter in the above mentioned bidirectional vector carrying mClover3 and H2B‐NIR FP, either of mCardinal, TagRFP658, or emiRFP2 was replaced by Purkinje neuron‐specific bidirectional promoter (E1b:4xcpce:E1b) enabling co‐expression of the corresponding NIR FPs with mClover3 predominantly in zebrafish cerebellar PCs while also inducing slight expression in tectal neurons of larval zebrafish.^44^

For generation of transgenic flies, the mCardinal, mMarron, iRFP‐VC, iRFP‐VC‐T2A‐HO1, miRFP2, and miRFP2‐T2A‐HO1 genes were codon‐optimized for expression in *D. melanogaster* using the online resource at http://www.bioinformatics.org/, synthesized de novo and cloned into the 20XUAS‐IVS‐Jaws‐mVenus_tr plasmid (Addgene #111553) by swapping the Jaws‐mVenus gene.

For expression in *C. elegans*, the genes of miRFP2, miRP2‐T2A‐HO1, and jGCaMP7s were codon‐optimized using SnapGene codon‐optimization tool, synthesized de novo by GenScript and cloned into an expression vector under the tag‐168 promoter the drives pan‐neuronal expression.

### 4.2 Protein purification and in vitro characterization

For protein purification, the phiLOV2.1, phiLOV3, TagRFP657, and TagRFP658 genes were cloned into pBAD‐HisD vector and transformed into TOP10 cells. To express the protein, bacterial cells were grown in RM medium supplemented with ampicillin and 0.002% arabinose for 15–18 hr at 37°C followed by 24 hr at 18°C. Proteins were purified using TALON Metal Affinity Resin (Clontech) and dialyzed overnight against PBS buffer, pH 7.4. To express miRFP720 and miRFP2 the genes were cloned in pBAD‐HisB plasmid and transformed into BW25113 bacterial cells, containing pWA23h plasmid encoding HO1 under rhamnose promoter. The colonies were grown in LB medium supplemented with ampicillin, kanamycin, 0.002% arabinose, and 0.02% rhamnose for 20 hr at 37°C. For protein purification, Ni‐NTA agarose (Qiagen) was used. Protein was eluted with PBS containing 100 mM EDTA, followed by dialysis overnight against PBS buffer, pH 7.4.

Spectral properties of the proteins were measured in PBS at pH 7.4. The fluorescence and absorption spectra of phiLOV2.1, phiLOV3, TagRFP657, and TagRFP658 were measured using a Fluorolog3 spectrofluorometer (Jobin Yvon) and a Lambda 35 UV/Vis spectrometer (PerkinElmer), respectively. The fluorescence and absorption spectra of miRFP720 and miRFP2 were measured with a CM2203 spectrofluorometer (Solar, Belarus) and NanoDrop 2000c spectrophotometer (Thermo Scientific), respectively. The extinction coefficient of phiLOV2.1 and phiLOV3 was determined as a ratio between the absorbance value of the peak at 374 nm, which correspond to FMN absorbance with extinction coefficient of 12,500 M^−1^·cm^−1^,^58^ and the value of the peak at major band peaked at ~476 nm. For determination of the quantum yield, integrated fluorescence of phiLOV3 was compared with that of equally absorbing phiLOV2.1, characterized by quantum yield value of 0.2.^31^ To determine extinction coefficients of TagRFP657 and TagRFP658, we relied on measuring the mature chromophore concentrations, using alkali‐denatured proteins as previously described.^32^ For determination of the quantum yield, integrated fluorescence of TagRFP658 was compared with that of equally absorbing TagRFP657, characterized by quantum yield value of 0.1.^32^ The extinction coefficient of miRFP720 and miRFP2 was determined as a ratio between the absorbance value of the peak at Q‐band and the value of the peak at Soret band, characterized by extinction coefficient of 39,900 M^−1^ cm^−1^.^8^ For quantum yield determination, the integrated fluorescence values of purified miRFP2 were compared with equally absorbing miRFP720 (quantum yield 0.061). pH titrations for phiLOV2.1, phiLOV3, TagRFP657, and TagRFP658 were performed in a 96‐well black clear bottom plate (Corning) by 1:20 dilution with a series of commercially available pH buffers (HYDRION) using a SpectraMax‐M5 plate reader (Molecular Devices) to read out fluorescence. To determine pH stability, miRFP2 and miRFP720 were diluted 1:100 into a series of home‐made pH adjusted buffers with NaOH and HCl (30 mM citric acid, 30 mM borax, or 30 mM NaCl) with pH values ranging from 3 to 10 in 0.5 pH units interval in a 96‐well black clear bottom plate (Thermo Scientific) using a Modulus II Microplate Reader (TurnerBiosystems) to read out fluorescence using 625 nm excitation and 660–720 nm emission filters. Size‐exclusion chromatography for phiLOV3 and TagRFP658 was performed by GenScript on a Superdex 200 10/300 GL column (GE Healthcare Life Sciences) using a gel filtration standard (#1511901; BIO‐RAD). Size‐exclusion chromatography for miRFP720 and miRFP2 was performed with a Superdex 200 10/300 GL column using GE AKTA Explorer (Amersham Pharmacia, UK) FPLC System.

Two‐photon spectrum and cross sections of TagRFP658 were measured in PBS buffer at concentrations ~1–5·10^−5^ M in 1 mm glass spectroscopy cuvettes (Starna cells) using an MOM two‐photon fluorescent microscope (Sutter Instrument) coupled with an Insight DeepSee (Newport) femtosecond laser, as described before.^33^ For the measurement of spectral shape, fluorescence was collected through a combination of 694SP (Semrock) and 630/60 (Chroma) filters for TagRFP658 and through HQ705/100 (Chroma) filter for Styryl 9‐M dye (Aldrich) in chloroform used as a reference standard. The two‐photon cross section was measured relatively to LDS 698 dye (Exciton) in chloroform54 at 1,150 nm using a combination of HQ705/100 (Chroma) and 630/60 (Chroma) filters and relatively to Rhodamine 700 in ethanol^59^ at 1,200 nm using 675/20 (Chroma) filter. The spectral shape was then scaled to these values. The differences between the peak absolute values obtained with two different standards were within 10%.

### 4.3 Protein characterization in mammalian cells

All procedures involving animals at MIT and Westlake University were conducted in accordance with the U.S. National Institutes of Health Guide for the Care and Use of Laboratory Animals and approved by the Massachusetts Institute of Technology or Westlake University Committee on Animal Care. HEK293FT (Invitrogen) and HeLa (ATCC CCL‐2) cells were maintained between 10 and 70% confluence at 37°C with 5% CO_2_ in DMEM medium (Cellgro) supplemented with 10% heat‐inactivated FBS (Corning), 1% penicillin/streptomycin (Cellgro), and 1% sodium pyruvate (BioWhittaker). Cells were authenticated by the manufacturer and tested for mycoplasma contamination to their standard levels of stringency and were here used because they are common cell lines for testing new tools. Primary mouse hippocampal neuronal culture was prepared as described previously. HEK293FT and HeLa cells were transiently transfected using TransIT‐X2 (Mirus Bio LLC) or calcium phosphate transfection kit (K278001, Invitrogen) according to the manufacturer's protocol and imaged 24–48 hr after transfection. Mouse primary hippocampal neurons were prepared from postnatal day 0 or 1 Swiss Webster (Taconic) mice (both male and female mice were used) and cultured as previously described.^33^ Neuronal cultures were transfected using calcium phosphate transfection kit (Life Sciences) according to the protocol described before.^33^ Transduction of neuronal culture was done at four DIV using ~10^9^ viral particles of rAAV8‐hSyn‐miRFP2 (Janelia Farm Viral Core, the rAAV genome titer was determined by dot blot) per well of standard 24‐well plate (Corning). Imaging of HEK293FT cells and neuronal cultures for Figures 1, 2, 3 was done using a Nikon Eclipse Ti inverted microscope equipped with a SPECTRA X light engine (Lumencor) with 475/28 nm and 631/28 nm exciters (Semrock), and a 5.5 Zyla camera (Andor), controlled by NIS‐Elements AR software, and using 10 × NA 0.3 and 40 × NA 1.15 objective lenses. Imaging of HEK293FT cells for Figure 3 was done under using a Nikon Eclipse Ti2‐E inverted microscope equipped with a SPECTRA III light engine (Lumencor) with 475/28 nm and 635/22 nm exciters (Semrock), a 680 nm solid‐state laser (MRL‐III‐680–800 mW, CNI Laser) and 680/13 nm exciter (Semrock), and a Orca Flash4.0v3 camera (Hamamatsu), controlled by NIS‐Elements AR software, and using 20× NA 0.75 objective lenses. To perform fair comparison of fluorescence intensity in Cy5 (excitation 635/22 nm from 637 nm laser, emission 730/140 nm) and Cy5.5 (excitation 680/13 nm from 680 nm laser, emission 710 LP) channels, images were acquired under matching excitation power (66 mW/mm^2^) and the same exposure time (100 ms).

NIH3T3 cells were grown in DMEM (4.5 μg/L glucose) supplemented with 10% FCS, and maintained in a 5% CO_2_ incubator at 37°C. The NIH3T3 stable cell line was generated by the transfection of pEF‐TGN46‐mScarlet‐iresPuro, followed by isolation and expansion of a clone grown in DMEM containing Puromycin at the concentration of 2 μg/ml. *Zebrafish PAC2 fibroblast cells* were cultured in L‐15 medium supplemented with 15% FCS, and maintained in an incubator at 28°C and atmospheric CO_2_. Transfection of plasmids was performed onto cells grown on μ‐slide 4‐well glass bottom dish (ibidi) using jetPRIME reagent (Polyplus) according to the manufacturer's protocol. For PAC2 cells, transfection was carried out in L‐15 medium supplemented with 5% FCS. Then, 6 hr after transfection, an equal volume of 15% FCS containing medium was added to adjust its final concentration of FCS at 10%. Live NIH3T3 and PAC2 cells were imaged 48 hr post transfection using a laser scanning confocal microscope (TCS SP8, Leica Microsystems, Germany) with 40× water (NA 1.1) objective. The BV solution (Sigma) at the final concentration 25 μM was added to PAC2 cells 48 hr after transfection, followed by recording of their images 3 hr after the BV administration. mClover3 was excited by an argon laser at 488 nm and detection range at 495–530 nm, whereas each of NIR RFs was excited by HeNe 633 laser and detection range at 660–785 nm. For three‐color imaging, transfected NIH3T3 cells were fixed with 4%PFA 36 hr after transfection, followed by imaging using a confocal microscope. mScarlet fluorescence was excited by a DPSS 561 nm laser with the detection range at 570–600 nm, followed by simultaneous imaging of mClover3 and the NIR FPs as described above.

### 4.4 In utero electroporation and acute slice preparation

Embryonic day (E) 15.5 timed‐pregnant female Swiss Webster mice (Taconic) were used for in utero electroporation as described before.^33^ The pAAV‐CAG‐emiRFP2‐P2A‐GFP or pAAV‐CAG‐mCardinal‐P2A‐GFP plasmid at ~1 μg/μl concentration were injected into the lateral ventricle of one cerebral hemisphere of an embryo. Acute brain slices were obtained from mice at P20–30 without regard for sex using standard techniques as described before.^33^ The brain slices were imaged using a Nikon Eclipse Ti2‐E inverted microscope equipped with a SPECTRA III light engine (Lumencor) with 475/28 nm and 635/22 nm exciters (Semrock), and a Orca Flash4.0v3 camera (Hamamatsu), controlled by NIS‐Elements AR software, and using 10× NA 0.45 and 20× NA 0.75 objective lenses.

### 4.5 Zebrafish larvae preparation and imaging

All experiments involving zebrafish at MIT and Technische Universität Braunschweig were conducted in accordance with protocols approved by Massachusetts Institute of Technology Committee on Animal Care following guidelines described in the U.S. National Institutes of *Health Guide for the Care and Use of Laboratory Animals* or by German legislation following European Union guidelines (EU Directive 2010_63) according to location of the respective experiments. Zebrafish larvae expressing TagRFP658 were prepared and imaged as described previously.^33^ Briefly, pTol2‐UAS‐zTagRFP658 plasmid was co‐injected with Tol2 transposase mRNA into homozygous nacre embryos of the pan‐neuronal expressing Gal4 line, tg(elavl3:GAL4‐VP16)nns6.^60^ Injected larvae were screened for NIR fluorescence in the brain and spinal cord at 2–3 days postfertilization (dpf) using the Nikon wide‐field microscope described above and subsequently imaged at 3–4 dpf using Zeiss Lightsheet Z.1 microscope. Lightsheets were generated by two illumination objectives (10×, NA 0.2), and the fluorescence signal detected by a 20× water immersion objective (NA 1.0). The laser line used for excitation was 638 nm. Optical filters used to separate and clean the fluorescence response included a Chroma T647lpxr as a dichroic, and a Chroma ET665lp. Tiled data sets were taken with the Zeiss ZEN Software, and subsequently merged and processed with FIJI, and Arivis Vision4D.

To co‐express the mCardinal, TagRFP658, and emiRFP2 H2B fusions with mClover3, one cell stage embryos of the pigmentation‐compromised zebrafish *brass* strain and *Tg(4xen.cpce‐E1B:gap‐mScarlet)* line were microinjected with the corresponding E1b:4xcpce:E1b expression vector described above together with *tol2* mRNA (1.5 nl of injection mix containing 25 ng/μl of both *tol2* and pTol2‐plasmid). mClover3 positive larval fish at 4 dpf were subjected to confocal microscopy analysis. The stable transgenic line, *Tg(4xen.cpce‐E1B: gap:mScarlet)* was generated by the injection of *tol1* mRNA together with a Tol1‐reporter plasmid in which membrane‐targeted mScarlet expression regulated by 4xcpce:E1b is restrictively induced in PCs, because ectopically expressed mScarlet in tectal neurons was eliminated by 4× miRNA181a target sequence inserted into the 3′UTR of the reporter gene.^44^ For membrane targeting, mScarlet was fused N‐terminally to the first 20 amino acids encoded by the zebrafish *gap43* gene. Fish larvae exhibiting no fluorescence in the corresponding channels were excluded from further imaging. Fluorescence imaging was performed using a laser scanning confocal microscope (TCS SP8, Leica Microsystems, Germany) with 40× water (NA 1.1) objectives. mClover3 (excited by an argon laser at 488 nm, detection range at 495–530 nm) and each of NIR FPs (excited by HeNe 633 nm laser, detection range at 660–785 nm) expressed in PCs were imaged simultaneously. For three‐color imaging, first, a DPSS 561 nm laser was used to excite mScarlet fluorescence, which was detected with the range at 570–600 nm, followed by simultaneous imaging of mClover3 and the NIR FPs as described above. Reconstructions and projections from z‐stacks of images were generated with the 3D‐projection program included in the LAS X software (Leica Microsystems, Germany). Acquired images were processed with FIJI to measure the fluorescent intensity ratio of each of NIR FPs and mClover3 in each PC. In vivo photostability of TagRFP658, emiRFP2, and mCardinal was assessed using PCs continuously exposed to HeNe633 laser set at 70% of the laser power in the software setting. The region of interest (116.25 μm × 116.25 μm) was drawn encompassing several PCs labeled with nuclear localized TagRFP658, or mCardinal, and single plan image (optical section: 3.56 μm) was taken every 0.648 s without interval for 5 min. Acquired images were processed with FIJI to measure the fluorescent intensity in each PC in each time point.

### 4.6 Preparation and imaging in drosophila

Transgenic fly lines with the following genotypes y1 w67c23; P{y[+t7.7] w[+mC] = UAS‐mCardinal} attP40, y1 w67c23; P{y[+t7.7] w[+mC] = UAS‐mMaroon} attP40, y1 w67c23; P{y[+t7.7] w[+mC] = UAS‐iRFP‐VC} attP40, y1 w67c23; P{y[+t7.7] w[+mC] = UAS‐iRFP‐VC‐T2A‐HO1} attP40, y1 w67c23; P{y[+t7.7] w[+mC] = UAS‐miRFP2} attP40, y1 w67c23; P{y[+t7.7] w[+mC] = UAS‐miRFP2‐T2A‐HO1} attP40 were generated by Bestgene using the user provided 20XUAS‐IVS plasmids described above. Flies were raised on standard cornmeal medium at room temperature. To drive pan‐neuronal protein expression, generated transgenic adult male flies were mated with C155‐GAL4 (pan‐neural, a gift from Littleton lab at MIT) virgin females to generate heterozygous (C155‐GAL4/+ or Y; Transgene/+).

Intact third instar larva and 2‐ to 4‐day old adult flies were immobilized on the coverslip for further imaging. To drive protein expression in specific neurons, generated transgenic flies were crossed with R84C10‐GAL4 to generate heterozygous progenies. Dissected brains from 5‐ to 6‐day old adult females were used for further imaging. No larvae or adult flies, carrying the genes of target proteins, were excluded from the study. The wide‐field Nikon microscope described above was used to acquire low magnification images and a Zeiss LSM 800 confocal microscope with Airyscan equipped with 631 nm laser for excitation for high magnification images.

### 4.7 *C. elegans* preparation and imaging

Worms were cultured and maintained following standard protocols.^61^ Transgenic worms with extrachromosomal array co‐expressing miRFP2 or miRFP2‐T2A‐HO1 with jGCaMP7s pan‐neuronally were generated by co‐injection of the two plasmids tag‐168::miRFP2 or tag‐168::miRFP2‐T2A‐HO1 with tag‐168::NLS‐jGCaMP7s into N2 background worms as described before.^62^ All plasmids were injected at 10 ng/μl. Worms exhibiting the highest green fluorescence in neurons were picked and mounted on 2% agarose pads on microscope slides, worms without green fluorescence were excluded from further imaging, immobilized with 5 mM tetramisole, covered by a coverslip, and imaged using a Zeiss LSM 800 confocal microscope with Airyscan equipped with 631 nm laser for excitation.

### 4.8 Data analysis and statistics

Data were analyzed offline using NIS‐Elements Advance Research software, Excel (Microsoft), OriginPro, ImageJ, the Microscope online application (https://www.fpbase.org/microscope), and Arivis Vision4D. Data collection for fluorescence spectra of NIR FPs was done using https://www.fpbase.org. All attempts at replication of the experiments were successful. We did not perform a power analysis, since our goal was to create a new technology; and as recommended by the NIH, “In experiments based on the success or failure of a desired goal, the number of animals required is difficult to estimate…” As noted in the aforementioned paper, “The number of animals required is usually estimated by experience instead of by any formal statistical calculation, although the procedures will be terminated [when the goal is achieved].” These numbers reflect our past experience in developing neurotechnologies. All attempts at replication of the experiments were successful. No randomization was used in the study. No blinding was used in the study.

## CONFLICT OF INTEREST

The authors declare no potential conflict of interests.

## AUTHOR CONTRIBUTIONS

**Kiryl D. Piatkevich** and **Edward S. Boyden**: Initiated the project and made high‐level designs and plans. **Kiryl D. Piatkevich** and **Erica E. Jung**: Developed the rapid directed molecular evolution approach. **Kiryl D. Piatkevich** and **Erica E. Jung**: Developed phiLOV3 and TagRFP658. **Kiryl D. Piatkevich** and **Siranush Babakhanova**: Developed miRFP2. **Kazuhiko Namikawa**: Designed and cloned emiRFP2. **Kiryl D. Piatkevich**: Characterized phiLOV3 and TagRFP568 in vitro and together with **Erica E. Jung** and **Hanbin Zhang** performed their characterization in mammalian cells. **Mikhail Drobizhev**: Measured two‐photon absorption properties in solution. **Lea Eisenhard** and **Kazuhiko Namikawa**: Performed protein characterization in NIH3T3 and PAC2 cells. **Erica E. Jung** and **Kiryl D. Piatkevich**: Performed electrophysiology experiments in cultured neurons. **Oksana M. Subach**, **Dmitry A. Korzhenevskiy**, **Tatiana V. Rakitina**, and **Fedor V. Subach**: Characterized miRFP2 in vitro. **Kiryl D. Piatkevich**, **Hanbin Zhang**, and **Siranush Babakhanova**: Characterized miRFP2 and emiRFP2 in mammalian cells. **Yangdong Wang**: Performed IUE. **Kiryl D. Piatkevich** and **Hanbin Zhang**: Performed acute brain slice imaging. **Kiryl D. Piatkevich**, **Erica E. Jung**, **Lea Eisenhard**, **Kazuhiko Namikawa**, and **Reinhard W. Köster**: Prepared zebrafish and performed their imaging. **Jing Shi** and **Hongyun Tang** prepared transgenic worms and **Xian Xiao** imaged them. **Kiryl D. Piatkevich**, **Siranush Babakhanova**, **Demian Park**, and **Wenjing Wang**: Prepared transgenic flies and performed their imaging. **Kiryl D. Piatkevich**: Performed the statistical analysis. **Kiryl D. Piatkevich**: Oversaw all aspects of the project, analyzed and interpreted the data, wrote the manuscript with contributions from all of the authors.

## Supporting information


**Appendix** **S1:** Supplementary Information

## Data Availability

All other data generated or analyzed during this study are available from the corresponding authors on reasonable request. The nucleotide sequences of the reported proteins are available at Genbank at the following accession codes: MZ682637 phiLOV3; MZ682638 TagRFP658; MZ682639 miRFP2; MZ682640 emiRFP2.
